# Mechanical Transitions in Crystals: The Low-Temperature Thermosalient Transition of a Mesogenic Polyphenyl

**DOI:** 10.1021/jacs.5c03448

**Published:** 2025-04-17

**Authors:** Emmanuele Parisi, Emanuela Santagata, Przemysław Kula, Jakub Herman, Sakuntala Gupta, Elena Simone, Salvatore Zarrella, Timothy M. Korter, Roberto Centore

**Affiliations:** † Department of Applied Science and Technology, Politecnico of Turin, I-10129 Turin, Italy; ‡ Department of Chemical Sciences, University of Naples Federico II, Via Cintia, I-80126 Naples, Italy; § Faculty of Advanced Technologies and Chemistry, Military University of Technology, 00-908 Warsaw, Poland; ∥ Department of Physics, Raiganj University, Uttar Dinajpur, 733134 Raiganj, W.B., India; ⊥ Department of Chemistry, Syracuse University, 111 College Place, 13244-4100 Syracuse, New York, United States

## Abstract

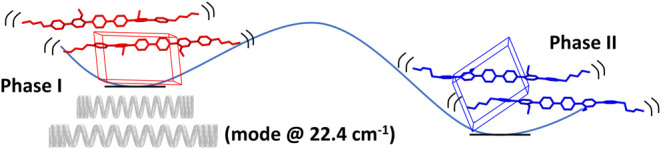

Thermosalient transitions are a subset of single-crystal-to-single-crystal (SCSC) transitions, in which the change of lattice parameters is highly anisotropic and very fast. As a result, crystals at the transition undergo macroscopic dynamical effects (hopping, jumping, and shattering). These transitions feature a conversion of heat to mechanical energy that can be exploited in the realization of advanced materials. Most thermosalient transitions are observed at temperatures higher than room temperature. Examples of low-temperature thermosalient transitions are rare. We describe a new example of a low-temperature thermosalient transition in a sexiphenyl compound. At about −40 °C, the parent single crystal (phase I) shatters into single crystal fragments of the new phase (phase II). The two phases have been studied by single-crystal X-ray analysis using a synchrotron source, variable-temperature Raman spectroscopy, and computational analysis of lattice normal vibration modes. A mechanism of the transition is proposed. We confirm colossal thermal expansion coefficients and supercells as reliable features of thermosalient transitions and add as a third feature a low-frequency principal optical vibration of the crystal lattice prompting the transition. Based on this, a roadmap for the automated prediction of thermosalient transitions in molecular crystals is also outlined.

## Introduction

Dynamic effects in molecular crystals are gaining increasing interest in view of potential applications in the realization of smart advanced materials.^1−3^ In many cases, these effects are thermally induced and are produced during a single-crystal-to-single-crystal (SCSC) transition. Depending on the features of the transition, the dynamic effects can be regular (e.g., reshaping, bending, twisting) or stochastic (hopping, jumping, violent shattering with emission of debris).^2^ The stochastic effects are generally produced when the SCSC transition is very fast (a displacive martensitic transition). In this case, the high mechanical stress accumulated inside the crystal because of the rapid transition is suddenly released in the form of dynamic effects. Such dynamic crystals (DCs) are called thermosalient.^1−4^ Although dynamic effects in crystal transitions have been sporadically reported over the years and in the early literature,^5^ the systematic study of DCs is recent: the first review of the subject is only ten years old.^1^ Nonetheless, a score of applications of these stimuli-responsive materials in smart devices has been reported, including soft and lightweight actuators, electric fuses, thermomechanically driven lifts, shape memory and ultra flexible single crystal electronic components, and energy storage devices.^6−9^ There are many open issues related with DCs, For instance, the comprehension of the mechanism of SCSC transitions producing the effects, the relation between the crystal structure of the parent and daughter phases, and the dynamic effects observed. Predictability of the dynamic effects, which, in turn, is related to the predictability of SCSC polymorphism, is another important open issue, perhaps the most important one.^10^

**Chart 1:**
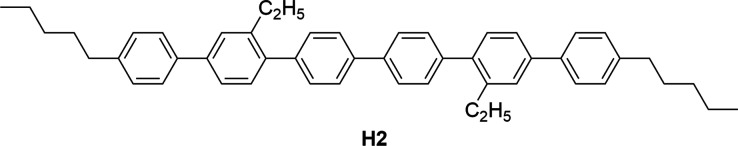
Chemical Diagram of the Compound Studied

From the side of applications of DCs, another relevant point is the temperature at which dynamic effects show up. In principle, it will be advisable to have a library of thermosalient crystals active in a broad range spanning from high to low temperatures. In most of the reported cases, DCs are active at temperatures higher than room temperature and in a few cases near/across room temperature.^1−3^ DCs active below room temperature are rare.^11−14^ Here, we discuss the low-temperature thermosalient transition (at −40 °C) of the sexiphenyl mesogenic compound shown in Chart 1. This compound, henceforth named **H2**, belongs to a class of compounds that have been recently synthesized and studied for applications in the field of very high birefringent liquid crystals for THz modulation in the telecommunication field (see the SI for the detailed description of the synthesis of **H2**).^15^

## Results and Discussion

**Figure 1:**
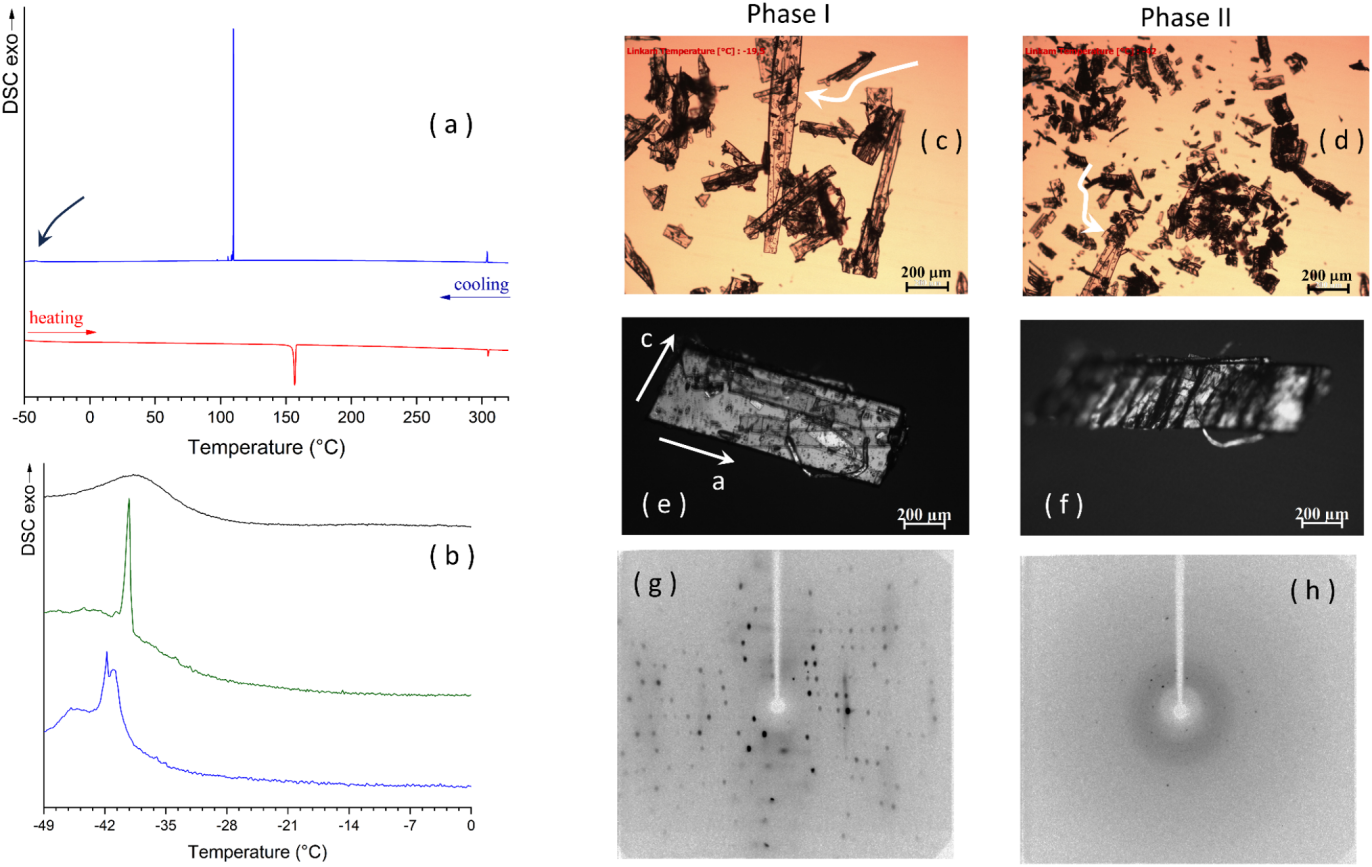
(a) DSC thermograms of **H2** (scan rate 2 K/min); (b) expansion in the low-temperature region of three consecutive DSC thermograms of **H2** on cooling after crystallization from the melt; (c) room-temperature polycrystalline sample of phase I with an arrow pointing to one big crystal; (d) same sample of (c) after partial transition to phase II, with the arrow pointing to the same crystal evidenced in panel (c) that is now rotated by about 180°; (e) single crystal of phase I, crystallized from acetone, with indication of unit cell axes; room temperature, crossed polarizers; (f) same crystal of (e) after the transition to phase II, crossed polarizers; (g) diffraction pattern (Mo Kα, 62 keV) of a single crystal of phase I at room temperature; and (h) diffraction pattern (Mo Kα, 62 keV), at room temperature, of a fragment of phase II isolated after the transition.

**H2**, crystallized from acetone or melt crystallized (phase I), melts at 155 °C to a nematic phase that isotropizes at 303 °C, Figure 1a. If a sample of phase I of **H2** is cooled, at about −40 °C, a solid-state transition to a new solid phase, named phase II, is observed, as evidenced by the exothermic peak in Figure 1a, b. This transition is monotropic, and by heating phase II, phase I is no longer obtained. The enthalpy change (Δ*H* = −0.9 kJ/mol) is comparable with other examples of thermosalient crystals.^5,16,17^

The transition from phase I to phase II of **H2** is thermosalient. At the transition, crystals of phase I, Figure 1c, undergo rapid movements, and afterward, they are shattered in single crystal fragments of phase II, Figure 1d and movie_1. Alternatively, they bend upward, taking at last a staircase shape and being fragmented into transversal single crystal slices, like the steps of the staircase, that can be recovered, Figure 1e, f and movie_2. Slight variations in the transition temperature, depending on the size and shape of the crystals are also observed, as reported for other thermosalient crystals.^4^ The single-crystal X-ray diffraction pattern of phase I can be collected with a conventional source, Figure 1g. In the case of phase II, the single crystal fragments recovered after the transition are small, and the diffraction pattern collected with a conventional source contains a few reflections, Figure 1h. Hence, data were collected with a synchrotron source at the Elettra synchrotron facility (Trieste, Italy).

**Figure 2:**
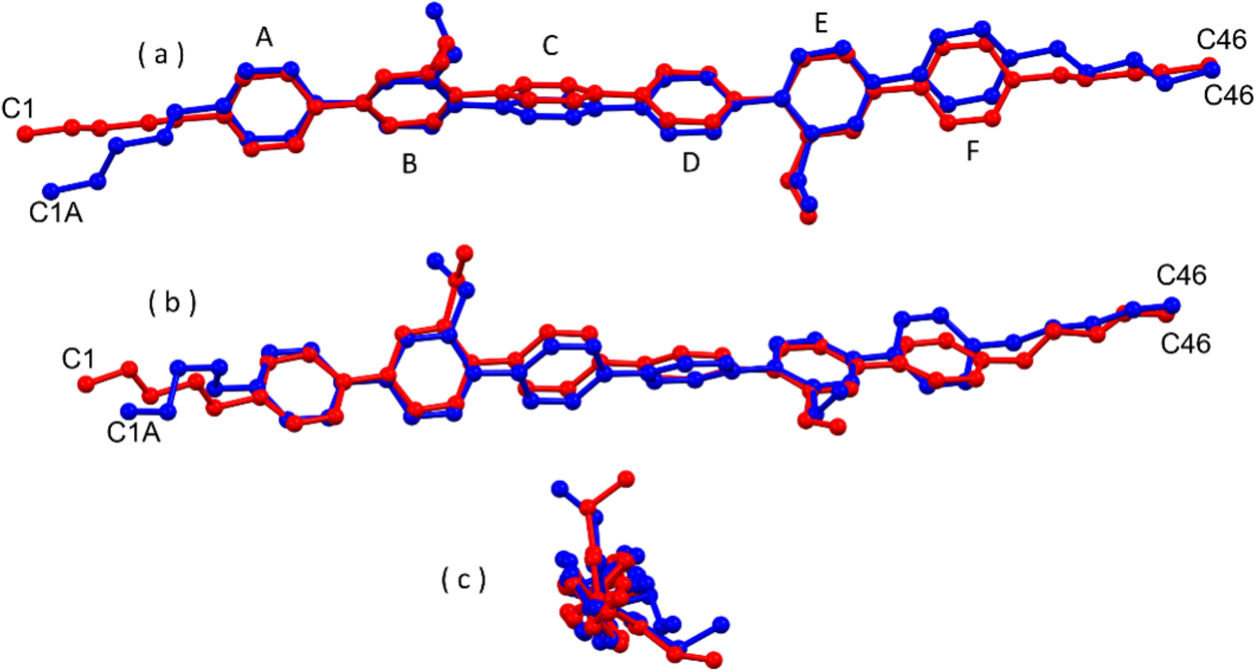
Superposition of the independent molecule of **H2** in phase I (red) and phase II (blue); H atoms omitted for clarity. (a, b) Two different face views; (c) a view along the long molecular axis. For phase II, only the more occupied split position of the disordered terminal chain is shown.

Crystal data of the two phases are reported in Table 1 (full data are reported in Table S1 of the SI).^18^ In Figure 2, we report a comparison of the X-ray molecular structures of **H2** in phases I and II by superimposing the crystallographically independent molecules. The independent molecules, with all phenyl rings connected in the para positions, have an elongated shape. The conformation is mainly determined by the dihedral angles between consecutive phenyl rings. The calculated dihedral angles between the mean planes of the six phenyl rings (A to F, see Figure 2a) are reported in the SI (Tables S2 and S3 for phases I and II, respectively), with a detailed discussion of the molecular conformation observed in the two phases.

**Table 1:** Some Crystal Properties of Phases I and II of **H2**

	phase I	phase II
*a* (Å)	9.900(4)	10.744(2)
*b* (Å)	12.346(7)	13.165(3)
*c* (Å)	17.154(7)	14.432(3)
α (°)	70.17(8)	104.27(3)
β (°)	77.86(5)	98.52(3)
γ (°)	81.59(5)	104.69(3)
*V* (Å^3^)	1921.8(19)	1864.6(7)
*T* (°C)	–15	–123
ρ (g/cm^3^)	1.132	1.166
Space gr.	*P*1̅	*P*1̅
*Z*, *Z*′	2, 1	2, 1

From Tables S2 and S3, it is evident that there is variability in the dihedral angles between consecutive rings. It is worth remembering here that for the biphenyl molecule, which can be considered the most simple model compound of **H2**, the conformation of minimum energy is not the planar one but one with a dihedral of 38° between the two rings, and conformations with dihedral angles between 0 and 60° are all within 1.1 kcal/mol from the minimum.^19^ The large and shallow minimum of the torsional energy centered at about 38° implies that crystal packing forces can easily induce changes in the dihedral angles in the range of 0–60°.

**Figure 3:**
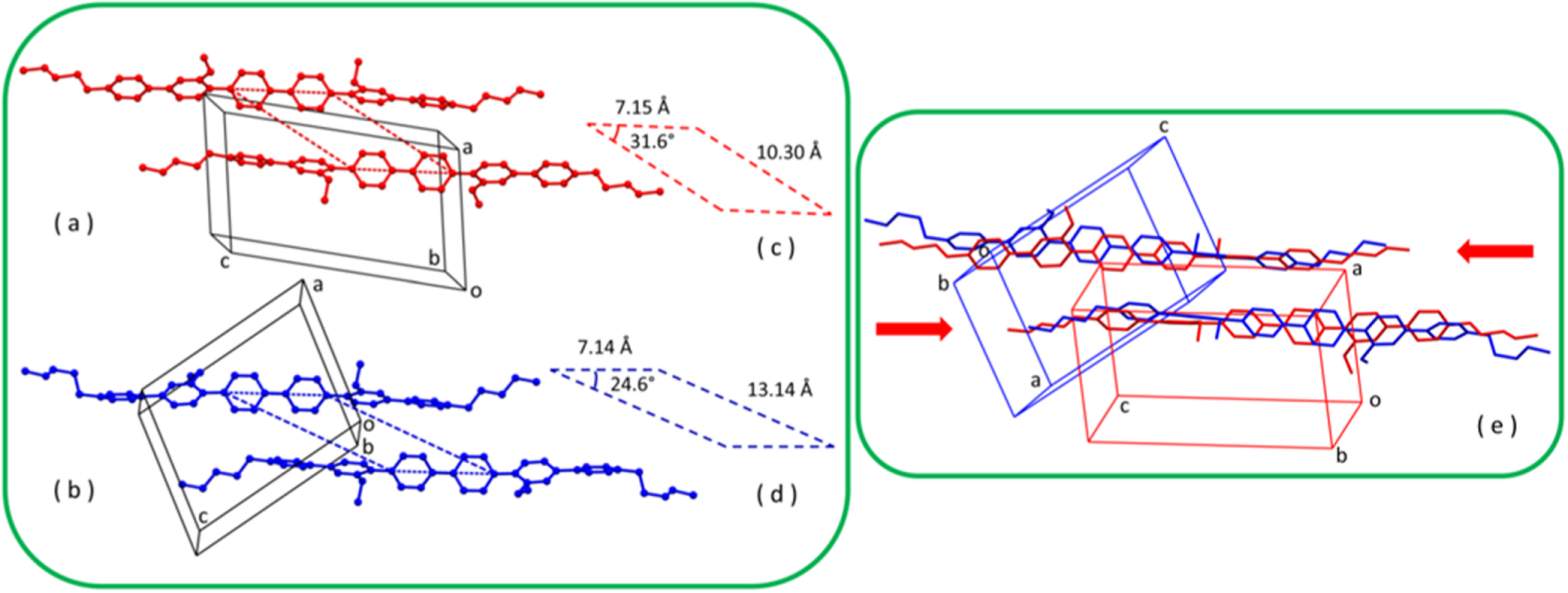
Correspondent views of the packing of phases I (a) and II (b) of **H2**, with parallelograms (c) and (d), showing the relative shifts of molecules; (e) superposition of the crystal structures of phase I (in red) and phase II (in blue) of **H2** in which two close centrosymmetrically related molecules are shown. The red arrows indicate the slipping movement of the two (red) molecules in going from phase I into phase II. H atoms omitted for clarity.

In Figure 3, a comparison of the packings of the two phases is reported, in which two centrosymmetrically related close molecules are shown. It is evident that molecules in the two phases keep their parallel orientation and basically also their relative position, this being consistent with the displacive, nondiffusive nature of the solid–solid transition.

Looking at Figure 3a,b, transition I→II can be viewed as a slipping of molecules in opposite directions parallel to their long axis, with simultaneous rearrangement of dihedral angles between phenyl rings. As further shown in Figure 3e, if we consider two centrosymmetrically related close molecules in phase I (colored in red), the two corresponding molecules in phase II (blue molecules) have slipped parallel to their long axes in opposite directions by about half the length of a phenyl ring (ca. 1.4 Å) while keeping the center of symmetry.

**Figure 4:**
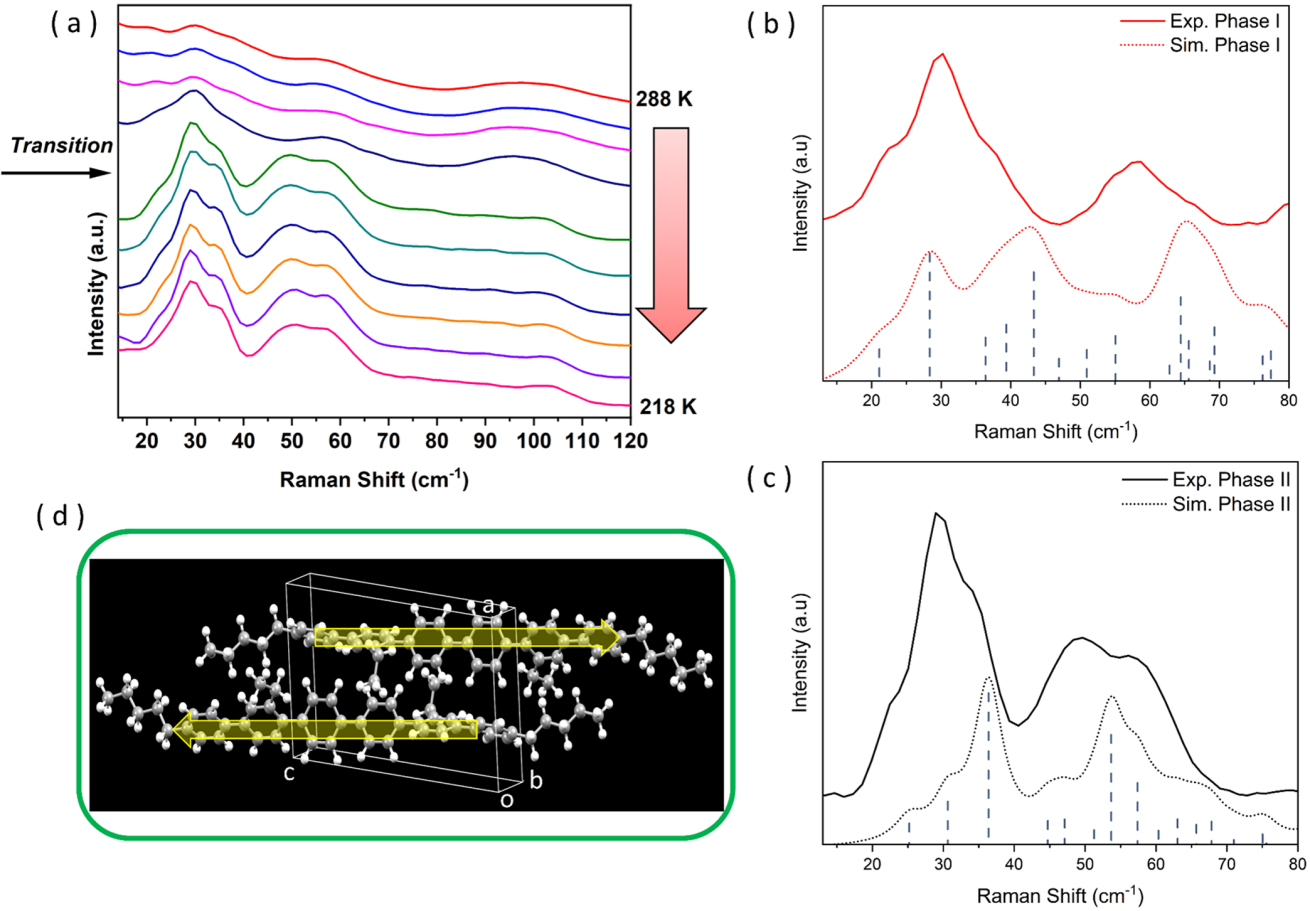
(a) Low-frequency Raman spectra of a single crystal of **H2** recorded on cooling from 288 to 218 K. The arrow marks the transition from phase I to phase II; (b) expansion of the low-frequency Raman spectra of **H2** in phase I at −15 °C and (c) in phase II at −30 °C. The calculated frequencies are reported as black vertical dashed lines whose height is proportional to the intensity; (d) Phase I, unit cell content with two arrows indicating the direction of the calculated autovector of the lowest frequency normal mode predicted at 21.103 cm^–1^ and observed at 22.4 cm^–1^.

These relative molecular displacements can be realized during Raman-active principal optical vibrations (*A*_g_ symmetry species) of the whole crystal lattice in the limit *k⃗*→0. In these vibrations, the displacements ξ⃗_*n*_ and η⃗_*n*_ of the barycenter of the two molecules from their equilibrium position in the *n*-th unit cell are given by the simple harmonic functions ξ⃗_*n*_ = *A⃗*cos(*ωt*), η⃗_*n*_ = *–A⃗*cos(*ωt*), with *A⃗* amplitude of the oscillation and *ω* (angular) frequency of the principal lattice vibration. To test this hypothesis, we have undertaken an experimental and computational analysis of the lattice vibrations of **H2**. Variable-temperature Raman spectra of **H2** were recorded from one single crystal in phase I cooled across the transition to phase II (see the SI) and are shown in Figure 4a.

There is strong a similarity between the spectra of the two phases for Raman shifts greater than 200 cm^–1^, which corresponds to the region of internal (i.e., intramolecular) crystal lattice vibration modes (see Figure S16 in the SI). External crystal lattice vibration modes, which correspond to oscillations of the whole molecules and are most sensitive to differences in the crystal packing, fall in the low-frequency region of the Raman spectrum, between 10 and 200 cm^–1^.^20^ In this region, the spectrum of phase II is different from phase I, and the transition is clearly evidenced in the set of spectra in Figure 4a. The centrosymmetric lattice oscillation that can induce the transition is expected in this low-frequency region. An expansion of the experimental spectrum in the region 10–80 cm^–1^ is reported in Figure 4b,c, alongside calculated frequencies of phase I and phase II in their stable states as solved by our diffraction experiments and not of the transient transition state connecting the two. The agreement between observed and calculated spectra is fair, and this allows a reliable assignment of vibrational motions in this region. The only exception to the good spectral correlation between theory and experiment is the peak predicted at 43.30 cm^–1^ in phase I, for which there is no clear assignment to experiment. The exact origin of this discrepancy is not clear, but the character of that vibration (terminal chain torsions) is unlike any other motions that exist in this frequency range (see Table S7 in the SI). If that single normal mode were ignored, the calculated spectra for both phases would be of similar quality. The equivalent vibration in phase II at 47.12 cm^–1^ (see Table S8 in the SI) is simulated with a low spectral intensity, which supports the interpretation that the mode intensity in phase I is overestimated.

Indeed, the simulated spectrum shows that the lowest frequency principal vibration of phase I, calculated at 21.103 cm^–1^ (0.632 THz) and observed at 22.4 cm^–1^ (0.671 THz), corresponds to a primarily translational motion with centrosymmetric molecules sliding along the *c*-axis in opposite directions (Figure 4d, movie_3), just as anticipated in Figure 3 by comparing the two crystal structures. This strongly supports the hypothesis that the vibration observed at 22.4 cm^–1^ can set off the I→II transition. A similar vibration is also present in phase II. It is calculated at 25.213 cm^–1^ (0.755 THz) and observed at 24.4 cm^–1^ (0.731 THz), so a lattice vibration could produce transition II→I as well (movie_4). This, however, does not happen for thermodynamic reasons because phase II has Gibbs free energy lower than I over the whole temperature range, confirming the monotropic nature of the transition (see Figure S19 in the SI). Altogether, our analysis suggests that the transition I→II can be induced not by random and uncorrelated thermal fluctuations as in the classic nucleation/growth mechanism^21^ but by a coherent elastic wave (lattice vibration/phonon) propagating across the whole crystal. This role of low-frequency lattice vibrations as the gateway for phase transitions has been recently discussed for some thermosalient and order–disorder transitions^22−26^ and witnesses the ever-increasing importance recognized to lattice dynamics in solid-state transformations.^25,27^

The activation of the monotropic transition I→II by cooling can also be explained in the framework of lattice dynamics. In fact, the principal lattice vibration responsible for the transition is at the bottom of the lattice vibration landscape (it is the lowest in frequency/energy). By lowering the temperature, the population of that specific phonon increases relative to all other phonons, and so the amplitude of that vibration responsible for the transition increases, as compared to all other principal vibrations.^21^

The analysis of linear thermal expansion coefficients (see the SI) indicates that phase I has one very high (colossal)^28,29^ thermal expansion coefficient (194·10^–6^ K^–1^). The corresponding principal axis has major components along crystallographic *a* and *b* axes, and so in directions basically transverse with respect to the direction of the long molecular axis, which is close to *c* for phase I. Additionally, linear thermal expansion coefficients of phase I are also anisotropic (the other two thermal expansion coefficients are −7.5 and 53·10^–6^ K^–1^). Colossal and anisotropic thermal expansion coefficients seem to be a signature of SCSC transitions, including thermosalient transitions.^10,30−35^

Additional information on the transition is obtained by viewing the crystal structures of phases I and II in a common reference system, and this can be achieved if a supercell common to both phases does exist. Suitable supercells of phases I and II are obtained by applying matrices *A*_*I*_ and *A*_*II*_ to the crystallographic unit cell vectors of Table 1, with
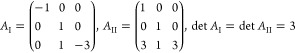
The parameters of the supercells are reported in Table 2.

**Table 2:** Supercells of Phases I and II of **H2** That Correspond to the Crystallographic Cells of Table 1^a^

	*a*′	*b*′	*c*′	α′	β′	γ′	*V*′
I	9.931	12.422	48.754	95.66	79.41	98.33	5834
II	10.744	13.165	46.656	97.00	61.20	104.69	5594

a Supercell parameters are in Å and °, and volume is in Å^3^.

**Figure 5:**
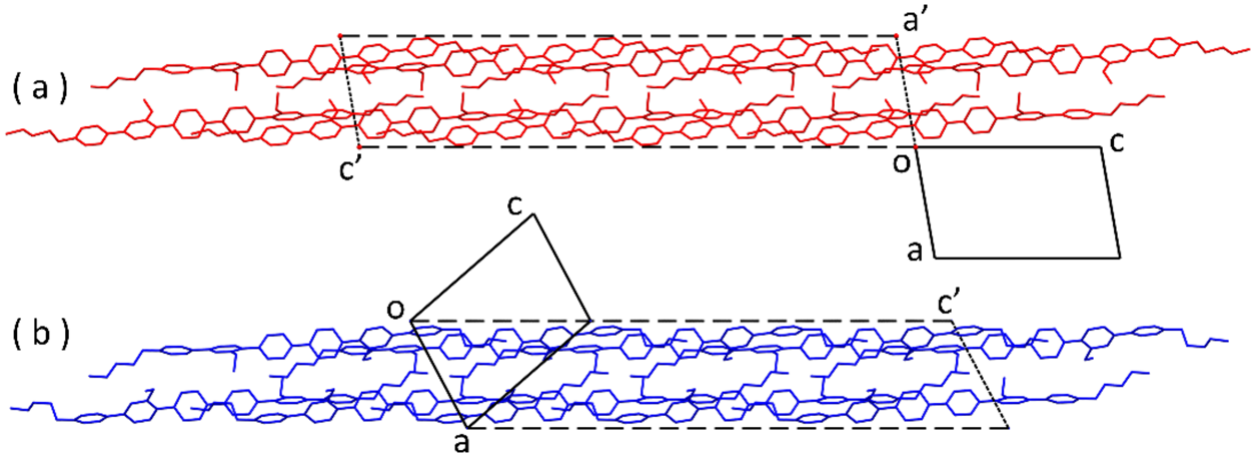
Crystal packing of phase I (a) and phase II (b) of **H2**, viewed along *b*. Crystallographic cells are drawn as solid black lines. Supercells of Table 2 are shown with dashed black lines. H atoms omitted for clarity.

The packings of phases I and II are shown in Figure 5. The strong metric similarity between the two phases is evident when looking at the supercells, and this, again, is consistent with the nondiffusive/displacive nature of the transition. The presence of a supercell common to both phases is considered another signature of SCSC transitions.^10^

From Table 2, at transition I→II on cooling, the changes in the supercell parameters are strongly anisotropic. In fact, the *c*′ axis undergoes a contraction (by about 5%), while *a*′ and *b*′ do expand (by 9 and 7%, respectively). This anisotropy can be responsible for the loss of integrity of single crystals during the transition. In fact, for single crystal specimens of phase I elongated along *c* (Figure 1c), the contraction of the *c*′ axis of the supercell, with *c*′ = *b⃗* – *3c⃗*, corresponds to a longitudinal compression of the crystals. Under these conditions, the linear shape of the crystals may become unstable (Euler’s elastic instability^36^), and a strong flection of the beam crystal is produced. This is consistent with the shattering of the slender crystals. For thicker crystals of phase I as that of Figure 1e, the compression is transverse (it is evident in Figure 1f), and this is consistent with the formation of transversal slices.^17,30^

So, after the analysis of crystal structures, lattice vibrations, and supercells, it seems that the SCSC thermosalient transition of **H2** has a strong flavor of a mechanical transition rather than a thermodynamic transition.^31,37^

## Conclusions

We have described a remarkable new example ofa low-temperature thermosalient transition, showing that the analysis of the crystal structures of the parent and daughter phases allows one to understand and predict the mechanical effects of the transition. We have also confirmed colossal thermal expansion coefficients and supercells as two features of thermosalient/SCSC transitions.^10^ Now, we propose a third indicator: a low-frequency principal optical vibration of the crystal lattice that can trigger the transition. We stress that supercells and low-frequency lattice vibrations are indicators that can be checked also for virtual (i.e., only computed) crystal structures. Based on this, a roadmap to the prediction of SCSC/thermosalient transitions for a given compound could rely on the determination of three sets of structures: *C*⊆*B*⊂*A*.^10^*A* is the set of potential polymorphs obtained after a crystal structure prediction task; *B* is the subset of potential polymorphs for which a common supercell exists; and *C* is the subset of potential polymorphs that are also related by a low-frequency principal optical vibration that serves as a gateway. We see no *a priori* hindrance to the automated implementation of this roadmap. In fact, a database of thermosalient crystals and a collection of reliable indicators are two features, which every possible procedure of automated prediction, based on Artificial Intelligence/Machine Learning^38^ or on conventional computing procedures, should rely on.

Our analysis also confirms the peculiar mechanical nature of SCSC/thermosalient transitions within the realm of solid-state transitions. This qualitatively different nature has been recognized and discussed, with different points of view, by several authors over the years.^10,23,25,31,39−44^ In our opinion, the increased amount of experimental data on these transitions, now available because of intensive research on dynamic crystals in the last years, calls for a rethinking of the whole matter of the mechanism of SCSC/thermosalient transitions with particular attention to the role of low-frequency lattice vibrations. After all, if a crystal can be considered as a supermolecule,^45^ crystal–crystal transitions should be considered as chemical reactions as well, and for chemical reactions, no universal mechanism exists.

## Supporting Information Available

A detailed description of the synthesis of **H2** with NMR and mass spectra; DSC analysis; full crystallographic and refinement details; powder diffraction patterns of phases I and II; analysis of molecular conformation in the two crystal polymorphs; thermal expansion data; experimental details of temperature-dependent Raman measurements; and full computational details for the optimization of the two crystal polymorphs and the calculation of lattice vibrations (PDF)
Transition I→II with shattering of single crystals of phase I in fragments of phase II (MP4)
Transition I→II with bending and staircase shaping (MP4)
Centrosymmetric molecules sliding along the *c*-axis in opposite directions, lattice vibration associated with transition I→II (MP4)
Lattice vibration possibly associated with transition II→I (MP4)
