# Supplementary material for: Mechanical Transitions in Crystals: The Low-Temperature Thermosalient Transition of a Mesogenic Polyphenyl
Source: J Am Chem Soc. 2025 Apr 17;147(17):14731–8. doi: 10.1021/jacs.5c03448 (PMC12046552; doi:10.1021/jacs.5c03448)
Supplement: Supplementary file 1 — ja5c03448_si_001.pdf [file ja5c03448_si_001.pdf]

Supporting Information file

Mechanical Transitions in Crystals: the Low Temperature Thermosalient Transition of a Mesogenic Polyphenyl

Emmanuele Parisi,[a] Emanuela Santagata,[b] Przemysław Kula,[c] Jakub Herman,[c] Sakuntala Gupta,[d] Elena Simone,[a] Salvatore Zarrella,[e] Timothy M. Korter,[e] Roberto Centore[b*]

[a] Department of Applied Science and Technology, Politecnico of Turin, I-10129 Turin, Italy.

[b] Department of Chemical Sciences, University of Naples Federico II, Via Cintia, I-80126 Naples, Italy.

[c] Faculty of Advanced Technologies and Chemistry, Military University of Technology, Warsaw, Poland.

[d] Department of Physics, Raiganj University, Raiganj, Uttar Dinajpur, Pin 733134, W.B., India.

[e] Department of Chemistry, Syracuse University, 111 College Place, Syracuse, New York, U. S. A. 13244-4100.

1. Experimental Part……………………………………………………………………………………..p. 2

2. Synthesis, chemical characterization, DSC analysis……………………………………………p. 2

3. X-ray Analysis………………………………………………………………………………………… p. 7

4. Conformational analysis……………………………………………………………………………..p. 10

5.Thermal expansion data……………………………………………………………………………..p. 12

6. Variable temperature Raman spectroscopy……………………………………………………p.17

7. Computational analysis……… ……………………………………………………………………..p. 19

8. References………………………………………………………………………………………………p. 51

1.Experimental Part

General. All reagents were analytical grade and were used without further purification. Transition points were determined by temperature controlled optical microscopy (Zeiss Axioskop polarizing microscope equipped with a Linkam PR600 hot stage) and DSC analysis (DSC 204 F1 Phoenix calorimeter (Netzsch, Selb, Germany), N_2 atmosphere, with the scanning rate 2 K/min on both the heating and cooling cycles and with the isothermal time of 5 minutes between cycles). NMR spectra were recorded with Bruker spectrometer operating at 500 MHz, in $CDCl_3$. The MS spectrum of **H2** was collected using a Shimadzu MS QP2010 instrument with Direct Inlet Probe (DIP-MS) with EI ionization, using the following temperature program of DIP probe: initial step 100 °C - 1 min, heating rate 20 K/min to final ramp 400 °C - 2 min). Single sublimation TIC peak was obtained, the mass spectrum was averaged from integrated peak.

2. Synthesis, chemical characterization, DSC analysis

2.1 Synthesis and chemical characterization

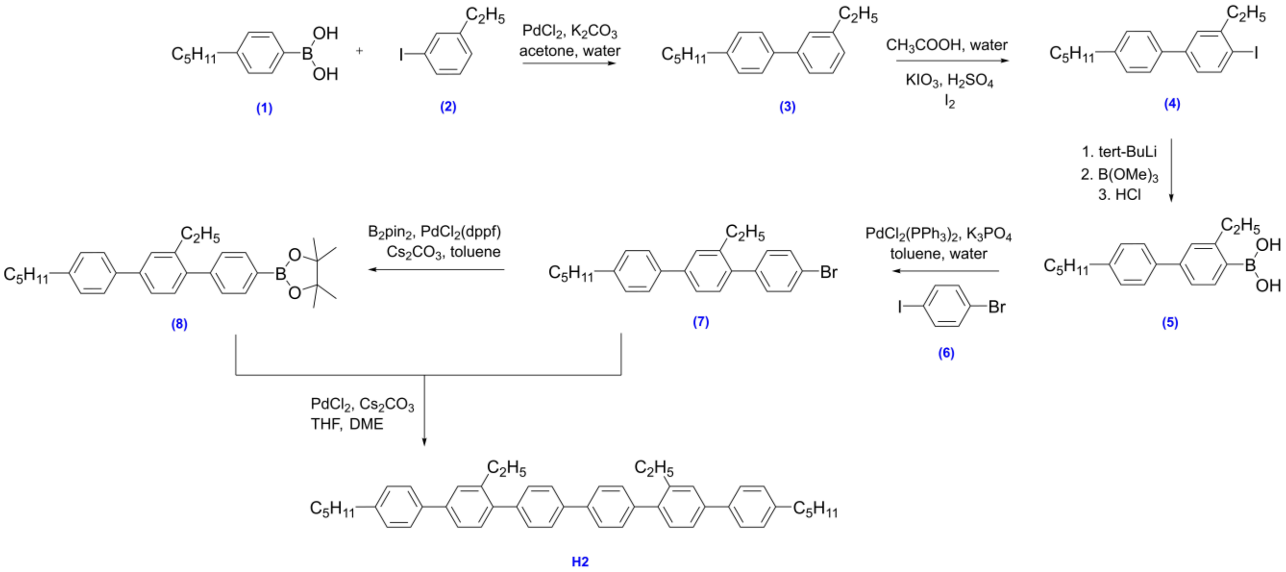

Scheme S1. Synthetic route to **H2** compound.

Experimental procedures

The synthetic intermediates and final compounds described here are, to the best of our knowledge, novel and not available in literature beyond our own work. Their synthesis and characterization were developed as part of our recent work.[1]

4'-pentyl-3-ethylbiphenyl (3). A flask containing 1-ethyl-3-iodobenzene **(2)** (20 g, 0.086 mol), 4-pentylbenzene boronic acid **(1)** (18 g, 0.094 mol), potassium carbonate (35.6 g, 0.26 mol), acetone (300 cm^3) and water (50 cm^3) was flushed with nitrogen under reflux for 30 min. Then catalyst $PdCl_2$

(3 mol%) was added and reaction was refluxed for 10 h. Next, reaction mixture was cooled to room temperature and poured into 5% HCl solution. The product was extracted with toluene, organic layer was washed with water, dried over $MgSO_4$. Solvent was evaporated, the product was distilled under reduced pressure: b.p. 130°C (0.1 mmHg), yield 17 g (78%).

4-iodo-4'-pentyl-3-ethylbiphenyl (4). A mixture containing 4'-pentyl-3-ethylbiphenyl **(3)** (17 g, 0.067 mol), I_2 (3.4 g, 0.013 mol), KIO_3 (5.5 g, 0.026 mmol), glacial acetic acid (500 cm^3), water (50 cm^3) and concentrated sulphuric acid (5 cm^3) was stirred for 3 h under reflux. After, the mixture was poured into Na_2SO_3 water solution. The product was extracted with toluene, organic layer was washed with water, dried over $MgSO_4$. The solvent was removed under reduced pressure. Product purified using column chromatography (silica gel – hexane). Brown oily liquid, yield 15 g (60%).

(4'-pentyl-3-ethylbiphenyl-4-yl)boronic acid (5). A flask containing 4-iodo-4'-pentyl-3-ethylbiphenyl **(4)** (15 g, 0.04 mol) and anhydrous THF (200 cm^3) was flushed with nitrogen and cooled to −78 °C in an acetone/dry ice bath. Solution of tert-butyllithium dissolved in pentane (0.088 mol) was added dropwise and temperature was kept below −70 °C for 2.5 h. Then, trimethyl borate (12.8 g, 0.088 mol) was added dropwise and temperature was kept below −70 °C. The reaction mixture was allowed to reach room temperature. Then, tetrahydrofuran was evaporated, and the crude product was treated with water and hydrochloric acid. Product was filtered off and dried. M.p=>230 °C (decomposition), yield 7.5 g (63%)

4-bromo-4''-pentyl-2'-ethyl-1,1':4',1''-terphenyl (7). The synthesis was performed using standard Suzuki cross-coupling procedure as described for **(3)**, using tripotassium phosphate and palladium(II)bis(triphenylphosphine) dichloride $PdCl_2(PPh_3)_2$. The product recrystallized from ethyl alcohol/acetone mixture (5/1 vol/vol), white crystals, yield 7 g (70%).

4,4,5,5-tetramethyl-2-(4''-pentyl-2'-ethyl-1,1':4',1''-terphenyl-4-yl)-1,3,2-dioxaborolane (8). A flask containing 4-bromo-4''-pentyl-2'-ethyl-1,1':4',1''-terphenyl **(7)** (7 g, 0.017 mol), bis(pinacolato)diboron (4.8 g, 18.9 mmol), cesium carbonate (19.6 g, 60 mmol) and toluene (200 cm^3) was flushed with nitrogen under reflux for 30 min. Then catalyst $PdCl_2$(dppf) (2 mol%) was added and reaction was refluxed for 10 h. Next, the reaction mixture was cooled to room temperature and poured into 5% HCl solution. The product was extracted with toluene, organic layer was washed with water, dried over $MgSO_4$. Solvent was evaporated. The product was recrystallized from acetone, yield 6 g (77%).

2’’’’,3’-diethyl-4,4’’’’’-dipentyl-1,1’:4’,1’’:4’’,1’’’:4’’’,1’’’’:4’’’’,1’’’’’-sexiphenyl (H2). The synthesis was performed using standard Suzuki cross-coupling procedure as described for **(3)**. Product was purified using liquid chromatography (silica gel-dichloromethane) and recrystallization from toluene, yield 1.0 g (45%), ^{1}H NMR (500 MHz, $CDCl_3$) δ ppm 0.95 (m, 6 H) 1.22 (t, J=7.48 Hz, 6 H) 1.39 (s, 4 H) 1.41 (d, J=3.66 Hz, 4 H) 1.70 (m, J=7.40, 7.40, 7.40, 7.40 Hz, 4 H) 2.69 (m, 4 H) 2.77 (q, J=7.53 Hz, 4 H) 7.30 (d, J=7.93 Hz, 4 H) 7.36 (d, J=7.63 Hz, 2 H) 7.48 (d, J=7.93 Hz, 4 H) 7.51 (dd, J=7.78, 1.68 Hz, 2 H) 7.60 (m, 6 H) 7.75 (d, J=8.24 Hz, 4 H). ^{13}C NMR (500 MHz, $CDCl_3$) δ 142.08; 142.00; 140.74; 140.43; 139.95; 139.20; 138.42; 130.45; 129.74; 128.81 ; 127.35; 126.95; 126.67; 124.28; 35.62; 31.59; 31.18; 26.37; 22.57; 15.77; 14.04. MS (EI): calcd. For $C_{50}H_{54}$ 654.98 $[M]^+$; found 655.00 $[M]^+$.

NMR spectra of **H2** are reported in Figures S1 and S2, mass spectrum in Fig. S3.

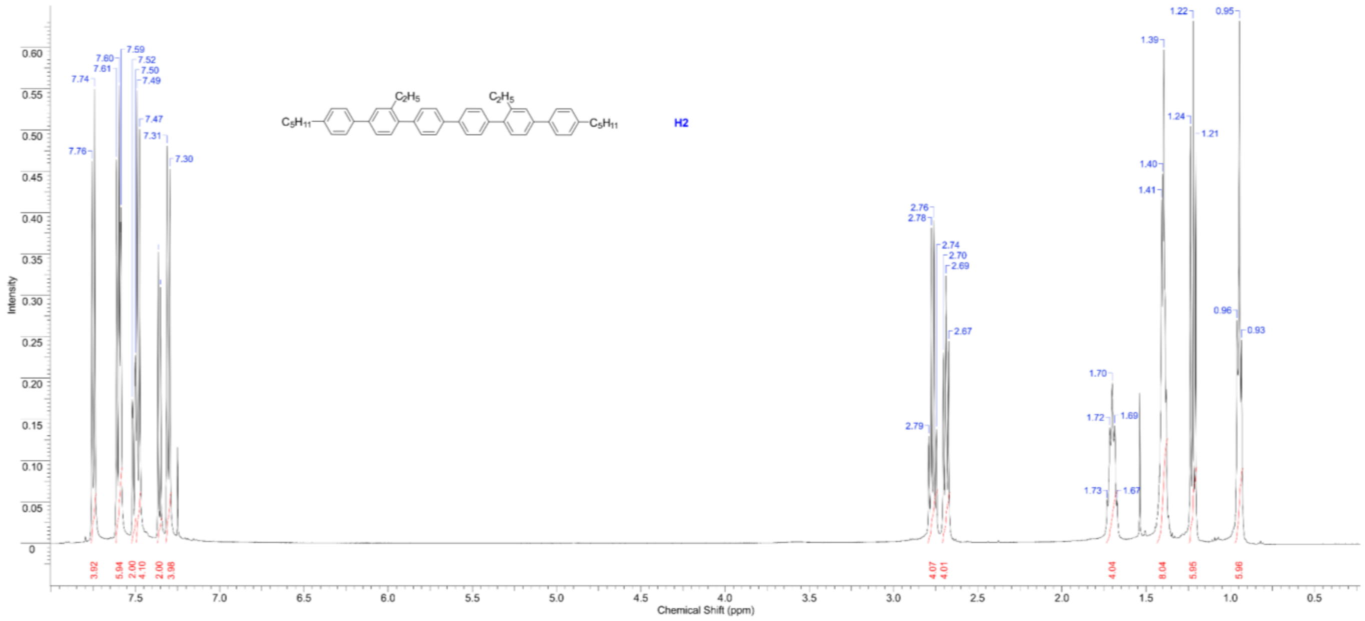

Fig. S1. ^{1}H NMR spectrum of **H2**.

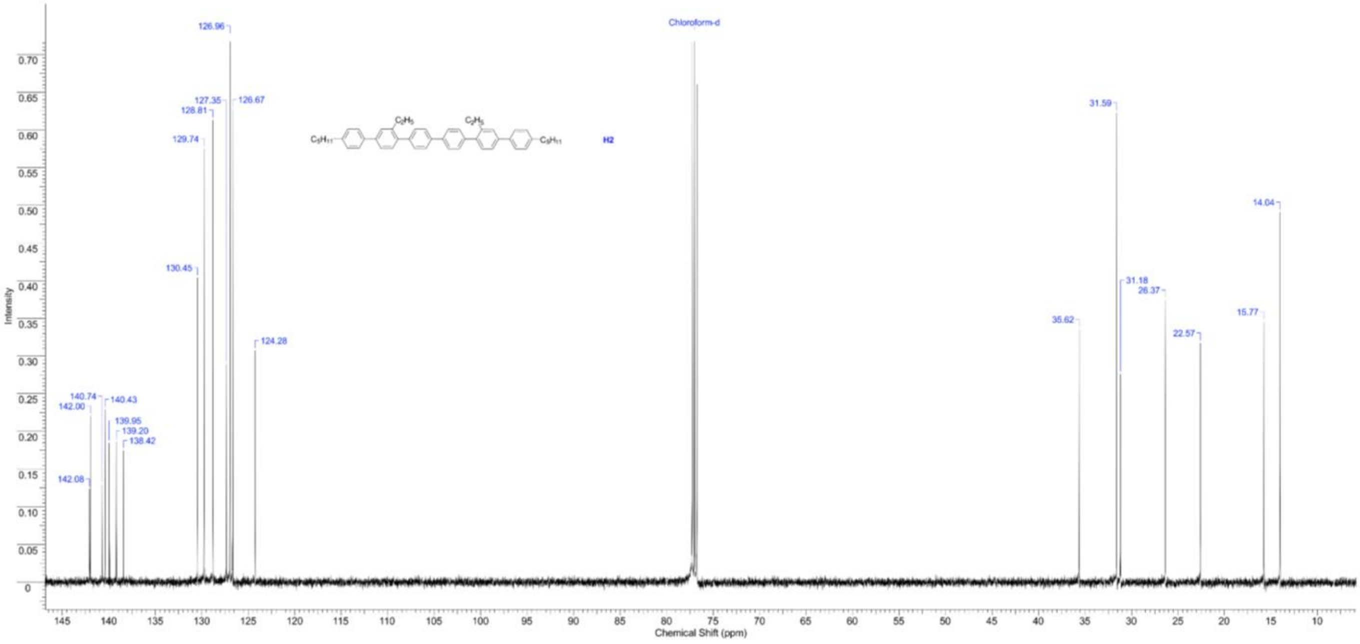

Fig. S2. ^{13}C NMR spectrum of **H2**.

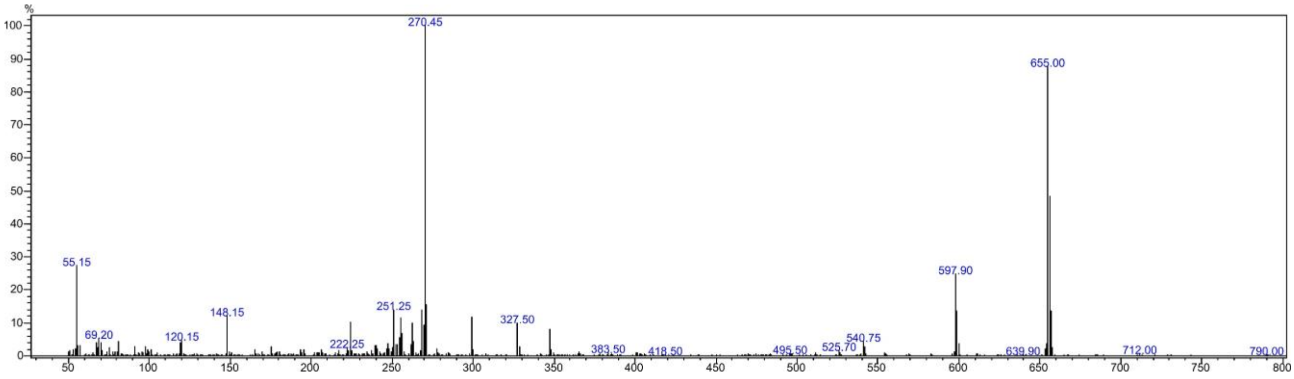

Fig. S3. Mass spectrum of **H2**.

2.2 DSC analysis

The low temperature thermosalient transition of **H2** was investigated at different cooling rates. The results are shown in Fig. S4.

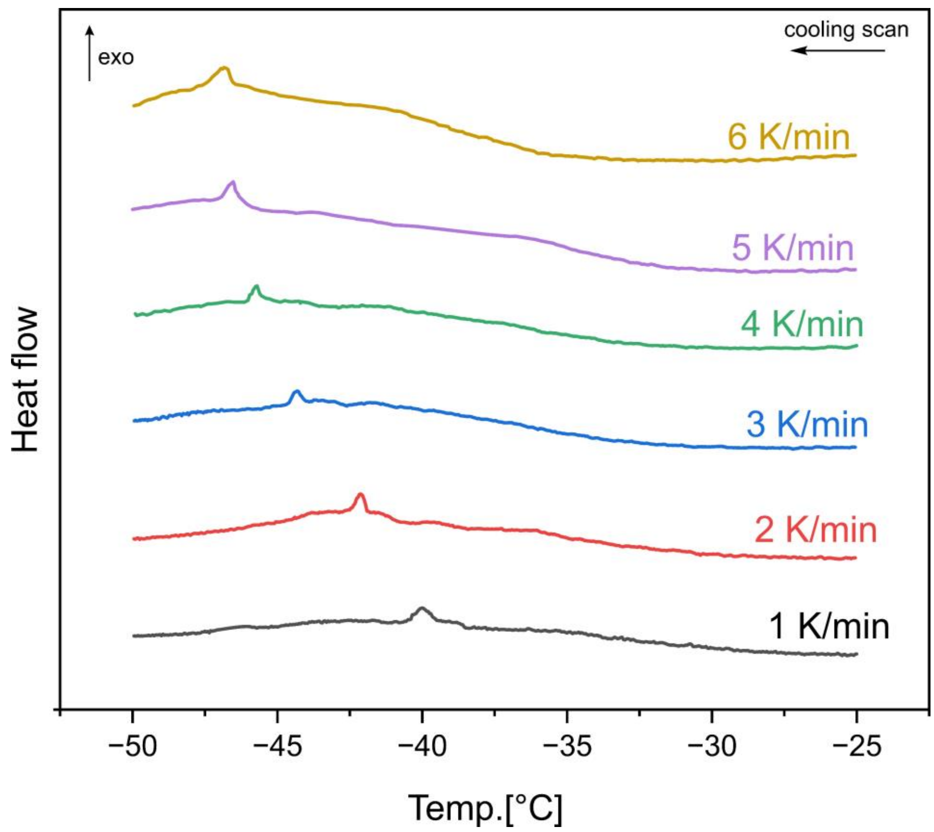

Fig. S4. DSC thermograms of **H2** at different cooling rates in the region of the thermosalient transition.

The Cr-Cr transition temperature shows a slight but consistent downward shift with increasing cooling rates in DSC measurements, because faster cooling leaves less time for atomic rearrangements, effectively requiring the system to “undercool” slightly before the transition can occur. The kinetic delay pushes the detectable transition to lower temperatures as cooling accelerates, while still maintaining the fundamental phase change. This cooling-rate dependence aligns with typical thermal hysteresis effects observed in solid-state transformations.

3. X-Ray analysis

3.1 Crystal preparation

Transparent prism-shaped colorless crystals of **H2**, suitable for X-ray analysis, were obtained by slow evaporation at 296 K from 50:50 (v:v) acetone-toluene solution.

3.2 Experimental data and refinement details

Data for single crystal structure determination of polymorph I of **H2** were measured on a Bruker-Nonius Kappa CCD diffractometer equipped with an Oxford Cryostream 700 apparatus, using graphite monochromated MoKα radiation (λ = 0.71073 Å). Reduction of data and semiempirical absorption correction were done using the SADABS program.[2] The structure was solved by direct methods (SIR97 program[3]). Single crystal X-ray diffraction data of phase II of **H2** were collected at the X-ray diffraction beamline (XRD1) of the Elettra Synchrotron (Trieste, Italy).[4] The NHV oil (Jena Bioscience, Jena, Germany) was used to dip and mount the crystal on the goniometer head with nylon loops (MiTeGen, Ithaca, USA). Data were collected at 150 K (nitrogen stream supplied through an Oxford Cryostream 700). The data were obtained using a monochromatic wavelength of 0.700 Å by the rotating crystal method on a Pilatus 2M hybrid-pixel area detector (DECTRIS Ltd., Baden-Daettwil, Switzerland). The diffraction data were integrated using XDS.[5] The crystal structure was solved in ShelXle[6] using ShelXT solution program by Intrinsic Phasing.

The structures were refined by the full-matrix least-squares method on F^2 using the SHELXL-2019/2 program[7] with the aid of the program WinGX.[8] H atoms were placed in calculated positions and refined by the riding model. For all H atoms, U_{iso} =1.2 × U_{eq} of the carrier atom was assumed (1.5 × U_{eq} for H atoms of methyl groups). The structure of polymorph I is affected by severe thermal motion in the two terminal pentyl chains, in particular in the chain C42 to C46. This is consistent with the fact that reflections beyond theta 25° were not observed and that the fraction of "observed" reflections, i. e. reflections with $I>2\sigma(I)$, is relatively small (3782 over 6659 total independent reflections). Attempts to model the disorder as static, with split positions, were unsuccessful. This strongly suggests the disorder be of dynamic nature. Some constraints were applied to bond lengths and anisotropic displacement parameters in the terminal chains. On the other hand, one lateral ethyl group is (statically) disordered. The disorder was modelled with two split positions, and the occupancy factor of the two positions was refined. All this accounts for the relatively high *R* factor in the final refinement of polymorph I. In polymorph II, one of the terminal pentyl tails is disordered. The disorder was modelled by two split positions (static disorder), whose site occupation factors were refined. Some constraints were applied on bond lengths, bond angles and anisotropic

displacement parameters. Full crystal and refinement data are summarized in Table S1. The analysis of the crystal packing was performed using the program Mercury.[9] CCDC 2421356-2421357 contain the supplementary crystallographic data for this article.

Tab. S1. Crystal, collection, and refinement data for the crystal structures described in the typescript.

	I	II
Chemical formula	$C_{50}H_{54}$	$C_{50}H_{54}$
Crystal system	Triclinic	Triclinic
Space group	$P\bar{1}$	$P\bar{1}$
Temp. (K)	258	150
a (Å)	9.900(4)	10.744(2)
b (Å)	12.346(7)	13.165(3)
c (Å)	17.154(7)	14.432(3)
α (°)	70.17(8)	104.27(3)
β (°)	77.86(5)	98.52(3)
γ (°)	81.59(5)	104.69(3)
V (Å^3)	1921.8(19)	1864.6(7)
Z, *Z'*	2,1	2, 1
λ (Å)	0.71073	0.700
μ (mm^{-1})	0.063	0.064
Meas., indep. reflns	22276, 6659	22064, 7027
R_{int}	0.0546	0.0307
θ_{max} (°)	24.995	25.387
R[I > 2σ(I)], *wR*(all)	0.0956, 0.3180	0.0628, 0.1777
Data/param./restraints	6659/470/12	7027/489/7
$\Delta\rho_{max}$, $\Delta\rho_{min}$ (e Å^{-3})	0.570/-0.424	0.531/-0.313
CCDC	2421356	2421357

3.3 Powder X-ray diffraction analysis

Powder X-ray diffraction (PXRD) measurements were performed using a PANalytical Empyrean X-ray diffractometer, equipped with Bragg–Brentano HD optics, a sealed tube copper X-ray source (Ni filtered CuKα radiation, λ = 1.54178 Å), fitted with a PixCel3D Medipix detector. The sample of phase I, in the form of as prepared powder, was loaded onto a silicon zero-background sample holder, and the diffraction pattern was recorded at room temperature over a 2θ range of 4° to 40° with a step size of 0.013° and an acquisition time of 100 s per step.

In Fig. S5 the PXRD pattern of phase I, alongside the powder pattern calculated from the single crystal X-ray structure, is reported. The matching between the experimental and calculated spectrum is good. This also indicates that the as prepared sample only contains phase I.

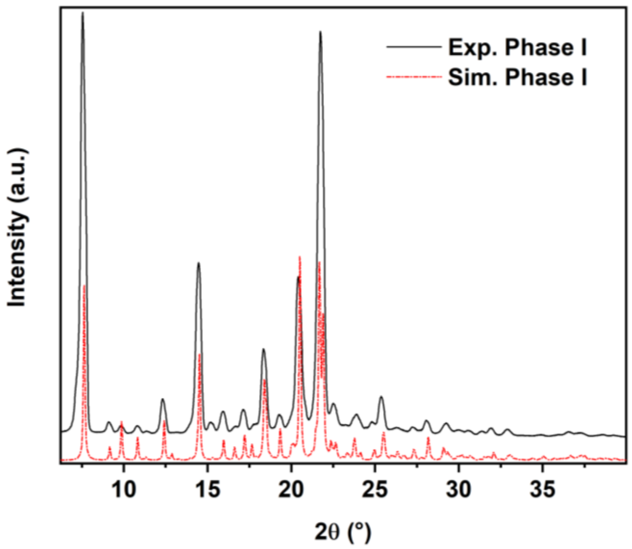

Fig. S5. Experimental PXRD pattern of phase I at 298 K. The simulated PXRD pattern of phase I calculated from the X-ray single crystal structure of phase I (at 258 K) is also shown.

After recording the PXRD pattern of phase I, the as prepared powder sample was put inside a vial that was cooled to low temperature, below -40 °C, by repeatedly dipping into liquid nitrogen, allowing the transition to phase II to take place. After, the sample was allowed to go to room temperature, and the PXRD pattern was recorded in the same conditions used for phase I. It is shown in Fig. S6 alongside the powder pattern calculated from the single crystal X-ray structure of phase II.

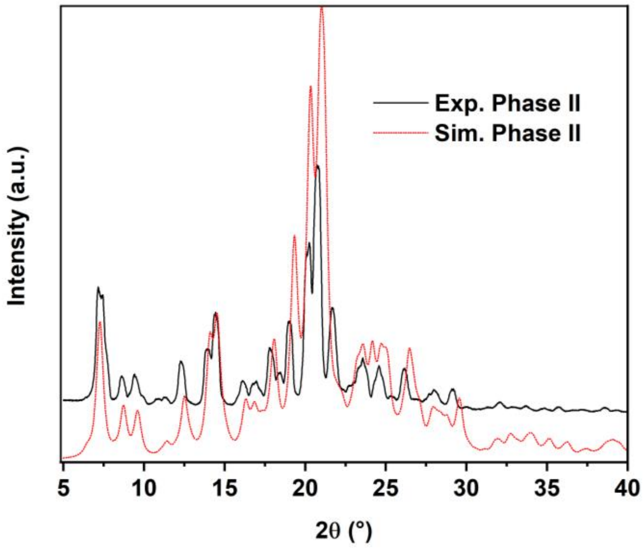

Fig. S6. Experimental PXRD pattern of phase II at 298 K. The simulated PXRD pattern of phase II calculated from the X-ray single crystal structure of phase I (at 150 K) is also shown.

Again, the matching between the experimental PXRD pattern and the pattern calculated from the X-ray single crystal structure of phase II is good.

4.Conformational analysis

For the commodity of the reader, we report again Fig. 2 (now Fig. S7) of the typescript. The dihedral angles between phenyl rings, calculated from the X-ray molecular structures, are reported in Tabs. S2 and S3.

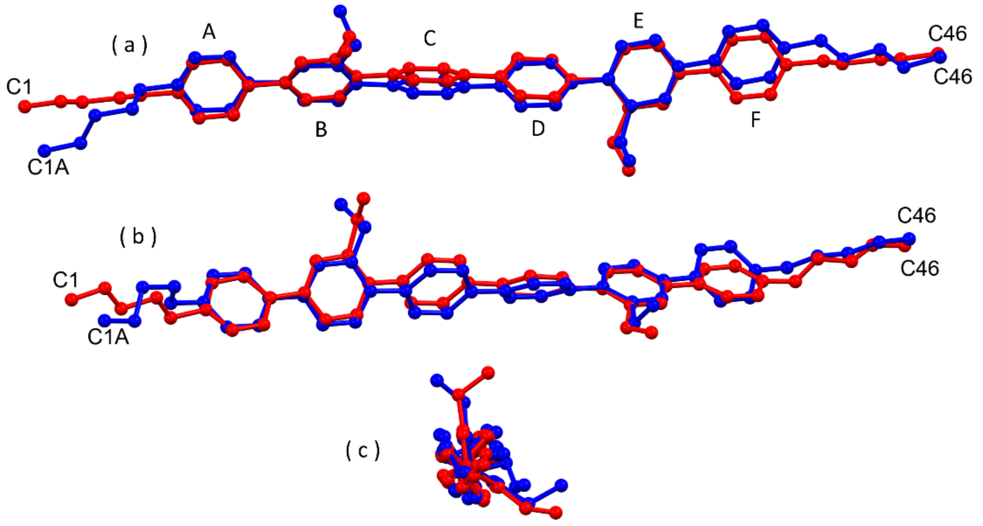

Fig. S7. Superposition of the independent molecule of **H2** in phase I (red) and phase II (blue); H atoms omitted for clarity. (a) and (b) are two different face view; (c) is a view along the long molecular axis. For phase II, only the more occupied split position of the disordered terminal chain is shown.

Table S2. Dihedral angles (°), with e.s.d.'s in parentheses, between the six phenyl rings of **H2** in phase I.

	A	B	C	D	E	F
A	0	29.83(7)	79.3(1)	73.3(1)	21.8(1)	25.8(2)
B	29.83(7)	0	49.9(1)	77.72(9)	50.38(8)	54.8(1)
C	79.3(1)	49.9(1)	0	27.84(7)	79.7(1)	75.3(1)
D	73.3(1)	77.72(9)	27.84(7)	0	51.9(1)	47.7(2)
E	21.8(1)	50.38(8)	79.7(1)	51.9(1)	0	4.5(3)
F	25.8(2)	54.8(1)	75.3(1)	47.7(2)	4.5(3)	0

Table S3. Dihedral angles (°), with e.s.d.'s in parentheses, between the six phenyl rings of **H2** in phase II.

	A	B	C	D	E	F
A	0	27.09(7)	74.50(7)	73.09(6)	26.98(6)	6.67(8)
B	27.09(7)	0	47.51(7)	79.99(6)	54.06(5)	29.35(7)
C	74.50(7)	47.51(7)	0	32.48(5)	78.56(6)	75.70(7)
D	73.09(6)	79.99(6)	32.48(5)	0	46.24(7)	72.35(7)
E	26.98(6)	54.06(5)	78.56(6)	46.24(7)	0	26.31(7)
F	6.67(8)	29.35(7)	75.70(7)	72.35(7)	26.31(7)	0

From Tables S2 and S3 it is evident that there is a large variability in the dihedral angles between consecutive rings. This variability is found in similar structures present in literature and retrieved from the CSD.[10] It is worthy to remember that, for the biphenyl molecule, that can be considered the most simple model compound of **H2**, the conformation of minimum energy is not the planar

one, but that one with dihedral of 38° between the two rings, and conformations with dihedral angles between 0° and 60° are all within 1.1 kcal/mol from the minimum (ref. 19 of the typescript). The large and shallow minimum of the torsional energy centered at about 38° implies that crystal packing forces can easily induce changes in the dihedral angles, in the range 0°-60°, and so solid-state polymorphism can be expected. In both phases, the largest dihedral angles between consecutive rings are those between rings (B, C) and (D, E) and the corresponding values are similar in the two phases. This can be related to steric effects due to the presence of the ortho ethyl group in rings B and E.

Another potentially relevant difference is the conformation of the terminal chains. In phase I the pentyl chains are in trans-planar conformation. In phase II, the pentyl chain attached to ring A, instead, is disordered over two split positions. The most populated one (77.9 %) has a gauche-type torsional angle around the central C-C bond of the chain, while the second position (22.1 %) corresponds to the most extended trans-planar conformation. In phase I the overall molecular length, defined as the distance between the two terminal C atoms, is 36.582 Å; in phase II it is 36.007 Å (1.6 % shorter). Also, the conformation of the ethyl group attached to ring B is different in the two phases.

5. Thermal expansion data

In Tab. S4 the unit cell parameters are reported as determined for phase I and II at different temperatures.

Table S4. Unit cell parameters at different temperatures for phases I and II of **He2**. Cell axes are given in Å, cell angles in °.

Phase	T (K)	σ_T (K)	a (Å)	b (Å)	c (Å)	α (°)	β (°)	γ (°)
I	300.0	2.0	9.938	12.439	17.161	70.4	78.01	81.75
I	285.0	2.0	9.931	12.442	17.162	70.4	77.94	81.67
I	280.0	2.0	9.926	12.407	17.158	70.44	77.95	81.64
I	275.0	2.0	9.918	12.397	17.153	70.48	77.9	81.6
I	265.0	2.0	9.915	12.381	17.154	70.49	77.86	81.53
I	260.0	2.0	9.913	12.37	17.155	70.49	77.86	81.5
I	255.0	2.0	9.909	12.363	17.153	70.51	77.82	81.46
II	240.0	2.0	10.841	13.21	14.405	104.62	99.06	104.63
II	220.0	2.0	10.81	13.195	14.4	104.57	98.85	104.78
II	200.0	2.0	10.8	13.2	14.491	104.45	98.7	104.77
II	180.0	2.0	10.755	13.158	14.43	104.35	98.66	104.81
II	150.0	2.0	10.744	13.135	14.432	104.27	98.52	104.69
II	100.0	2.0	10.687	13.125	14.391	104.15	98.37	104.52

From data of Tab. S4, the phase transition I → II between 255 K and 240 K is evident.

The experimental values of Tab. S4 have been analyzed by using the program PAScal,[11] that allows to calculate the coefficients of linear thermal expansion, α_i, along the principal axes and the coefficient of linear thermal expansion of the volume. The coefficients are defined as:

$$\alpha_i = \frac{1}{l_i}\left(\frac{\partial l_i}{\partial T}\right)_P, \alpha_V = \frac{1}{V}\left(\frac{\partial V}{\partial T}\right)_P$$

The coefficients of phase I, calculated using data of Tab. S4 from 255 K to 300 K, are reported in Table S5, with estimated standard deviations in parentheses.

Table S5. Linear coefficients of thermal expansion of phase I.

Principal axis	α_i/10^{-6} K^{-1}
X_1	-7.5(9)
X_2	53(4)
X_3	194(17)
V	241(20)

Plots of relative change in principal axis length, as a function of temperature, are reported in Fig. S8.

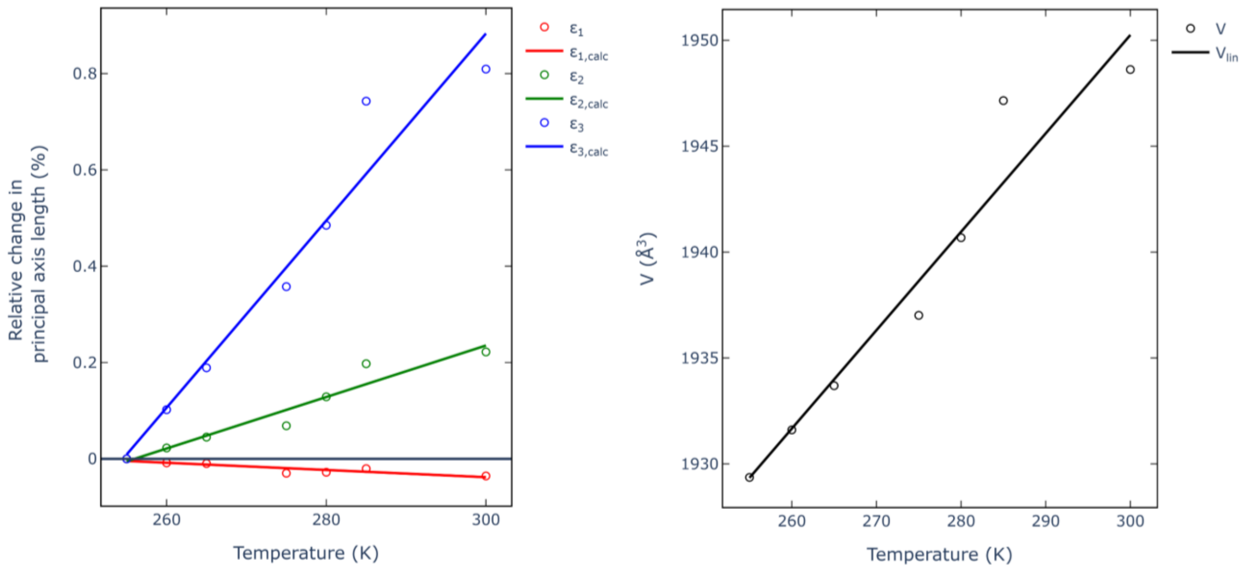

(a) (b)

Fig. S8. (a) Relative change in principal axis length for phase I of **H2**; (b) Linear fit of experimental cell volume for phase I of **H2**.

The principal axes of Tab. S5 are expressed in terms of vectors of the unit cell (***a, b, c***) according to the following relations:

$$\begin{cases} X_1 = 0.4535\boldsymbol{a} - 0.2107\boldsymbol{b} + 0.866\boldsymbol{c} \\ X_2 = \ 0.8468\boldsymbol{a} + 0.4385\boldsymbol{b} - 0.3011\boldsymbol{c} \\ X_3 = -0.5942\boldsymbol{a} + 0.8028\boldsymbol{b} + 0.0495\boldsymbol{c} \end{cases}$$

The thermal expansion behavior of phase I shows a high anomalous value of α_3. The corresponding autovector has components along $\boldsymbol{a}$ and $\boldsymbol{b}$ axes, with very small component along $\boldsymbol{c}$, as is also evidenced in the shape of expansivity indicatrix, for which two projections are shown in Fig. S9.

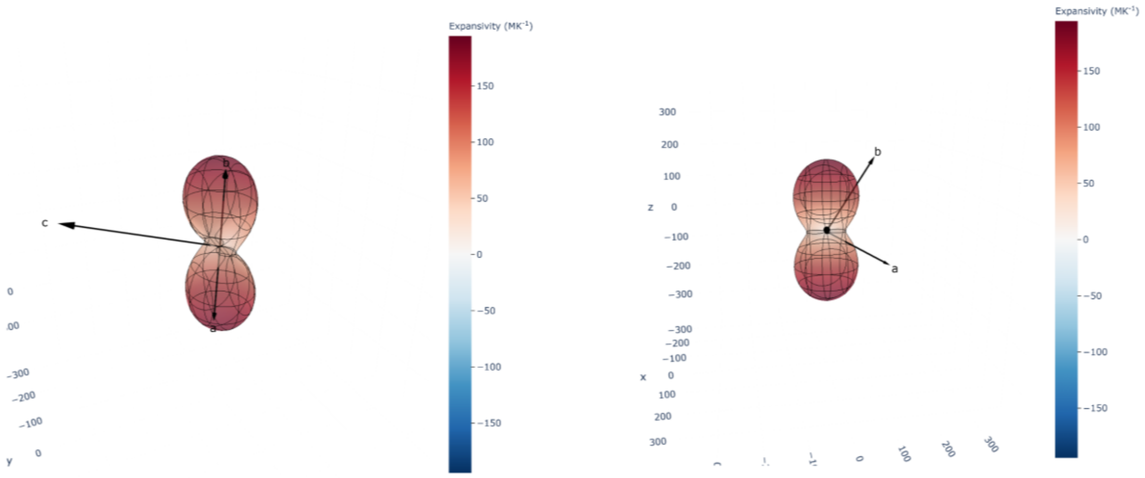

Fig. S9. Indicatrix of thermal expansivity of **H2** (phase I) viewed along the unit cell axes.

The directions of the three autovectors of Tab. S5 are reported, within the unit cell, in Fig. S10.

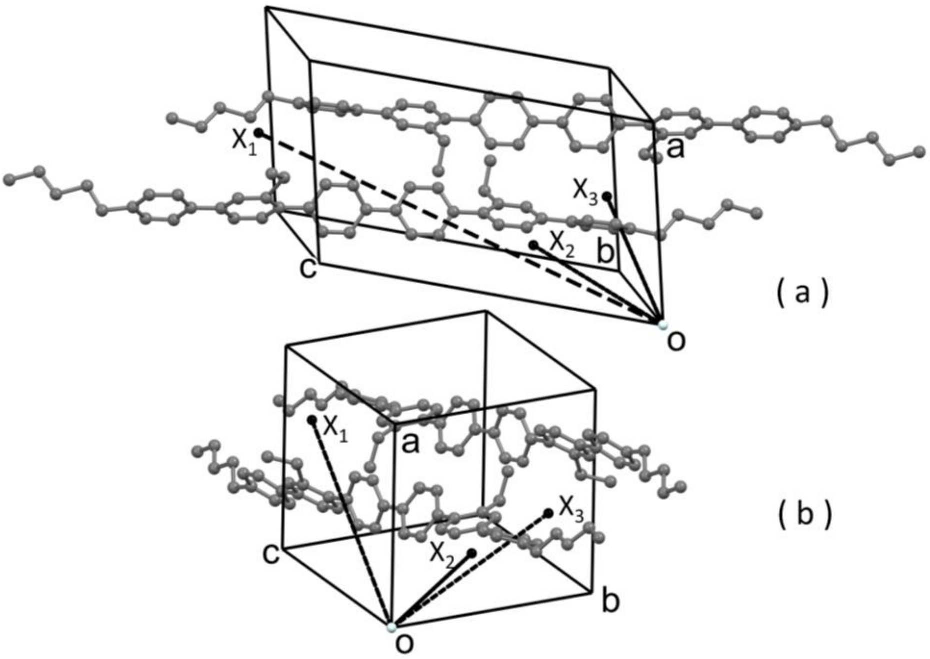

Fig. S10. Direction of the principal axes of thermal expansion of phase I (X_1, X_2, X_3), showed as dashed black lines, in two different views of the unit cell, (a) and (b).

From Fig. 10 it results that X_1 is mainly longitudinal while X_2 and X_3 are basically transverse with respect to the direction of the long molecular axis. So, the overall thermal expansion of phase I is anisotropic, consisting of transverse expansions coupled with a small longitudinal contraction.

The coefficients of phase II, calculated using data of Tab. S4 from 100 K to 240 K, are reported in Table S6, with estimated standard deviations in parentheses.

Table S6. Linear coefficients of thermal expansion of phase II.

Principal axis	$\alpha_i/10^{-6}$ K^{-1}
X_1	-55(9)
X_2	58(5)
X_3	111(4)
V	113(10)

Plots of relative change in principal axis length, as a function of temperature, are reported in Fig. S11.

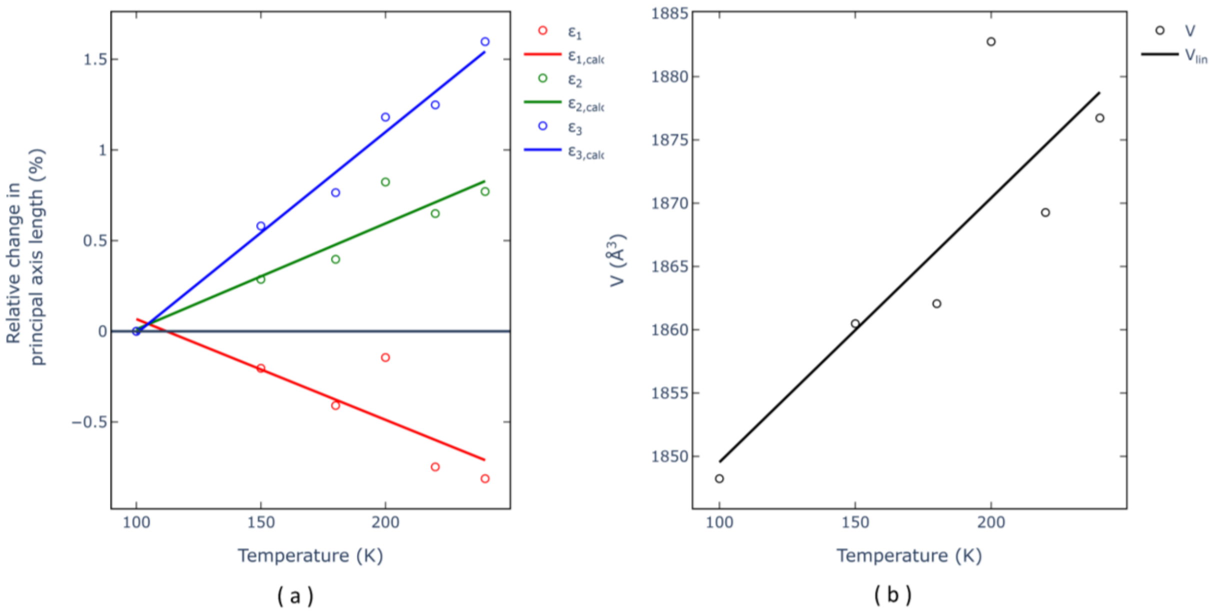

Fig. S11. (a) Relative change in principal axis length for phase II of **H2**; (b) Linear fit of experimental cell volume for phase II of **H2**.

The principal axes of Tab. S6 are expressed in terms of vectors of the unit cell (***a, b, c***) according to the following relations:

$$\begin{cases} X_1 = 0.5876\boldsymbol{a} + 0.6202\boldsymbol{b} + 0.5197\boldsymbol{c} \\ X_2 = -0.0274\boldsymbol{a} + 0.7337\boldsymbol{b} - 0.6789\boldsymbol{c} \\ X_3 = 0.9369\boldsymbol{a} - 0.2181\boldsymbol{b} - 0.2731\boldsymbol{c} \end{cases}$$

The thermal expansion behavior of phase II shows a high value of α_3. The negative value of α_1 has also to be remarked. Two projections of the expansivity indicatrix are shown in Fig. S12.

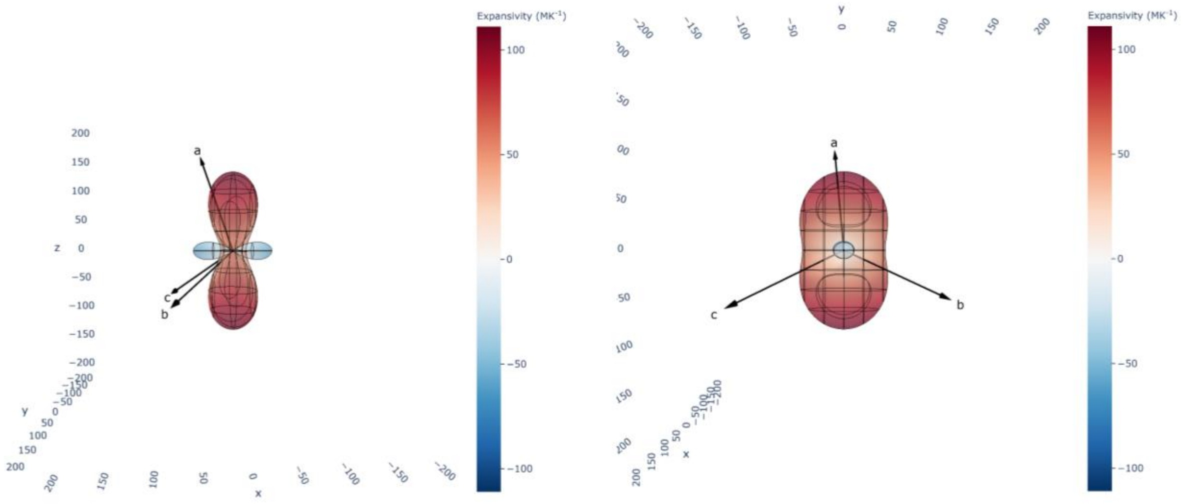

Fig. S12. Indicatrix of thermal expansivity of **H2** (phase II) viewed along the unit cell axes.

In Fig. S13, the directions of the three autovectors of Tab. S6 are reported within the unit cell.

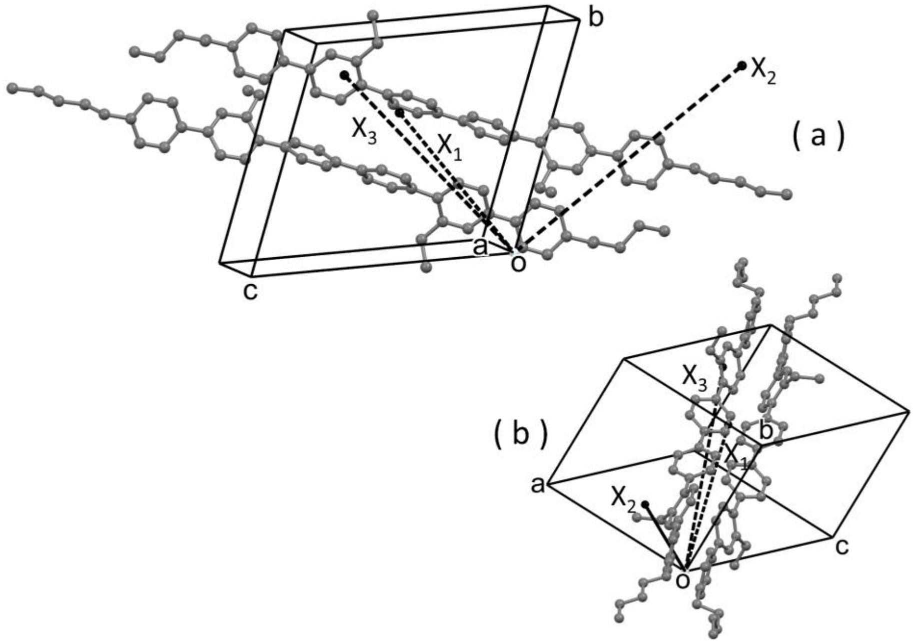

Fig. S13. Direction of the principal axes of thermal expansion of phase II (X_1, X_2, X_3), showed as dashed black lines, in two different views of the unit cell, (a) and (b).

From Fig. S13 it results that X_1, X_2 and X_3 have significant components both in longitudinal and transverse directions. The overall thermal expansion is again anisotropic.

From data of Tab. S4 the volume of the unit cell as a function of the temperature can be evaluated over the entire temperature range that was investigated. The data are reported in Fig. S14. A linear fit of the data of each phase is reported in the Figure only at indicative level. The transition at about 250 K is clearly evidenced by the sudden change in the slope of the linear fits.

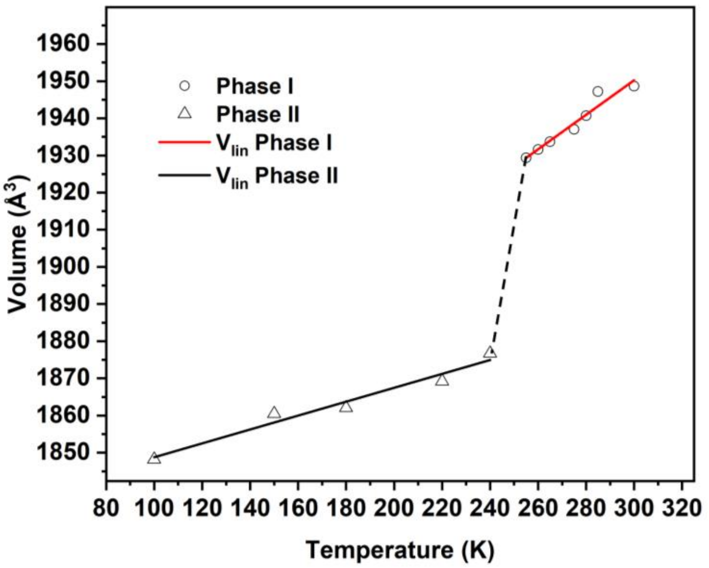

Fig. S14. Comparison of the linear fit of experimental cell volume for phase I and phase II of **H2**.

6. Variable temperature Raman spectroscopy

6.1 Experimental part

Variable temperature Raman spectra of phases I and II were acquired using a 633 nm laser source with a LabRAM HR Evolution spectrometer (HORIBA Scientific, France). The incident laser beam was focused on the sample by a 50xLWD objective (Olympus) with a numerical aperture of 0.50. Backscattered radiation was collected with a Synapse Plus BIDD Detector (1024 x 256 pixels), utilizing a 600/nm grating. ULF-633 filter was applied in order to allow measurements covering both Stokes and Anti-Stokes sides of the spectrum. Elemental sulfur was used for calibration in the low wavenumber region. Thelaser power was set at 100%. Spectra were acquired with a 1-second acquisition time for 15 accumulations. Temperature ramp measurements were performed with Linkam THMS600 heating stage, connected to a liquid nitrogen pump (Linkam Scientific Instruments, UK). The sample, in the form of a single crystal, was loaded inside the heating stage under nitrogen flow and the temperature was then varied within the LabSpec suite software in the range between -55 and 25 °C with heating and cooling rate of 10 °C/min. The sample was focused again after the phase transition.

6.2 Results

In Fig. S15 and S16 Raman spectra are reported in detail.

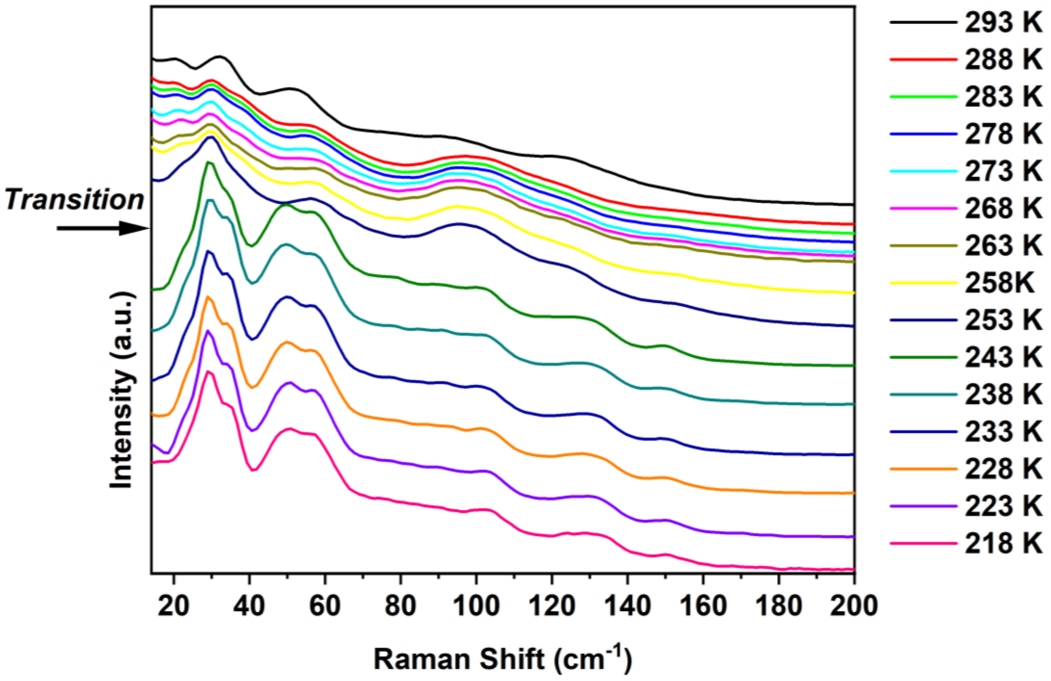

Fig. S15. Raman spectra of **H2** taken on cooling at temperature intervals of 5 K starting from 293 K down to 218 K. The transition from phase I to phase II is evident between 253 and 243 K.

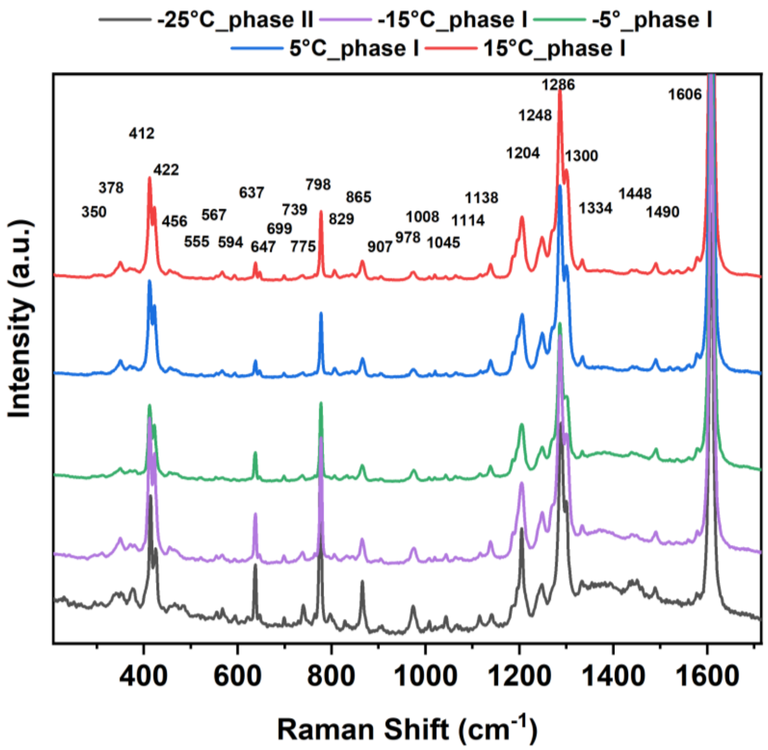

Fig. S16. Raman spectra of phase II of **H2** at different temperatures (15 °C, 5 °C, -5 °C, -15 °C) and of phase II at -25 °C.

The spectra look very similar in peak positions and relative intensity in the region 400-1700 cm^{-1}, which corresponds to internal lattice vibration modes. This is expected because molecular structure is similar in phases I and II. On the other hand, in the region below 200 cm^{-1}, corresponding to external lattice vibration modes, the spectra of the two phases are different.

7. Computational analysis

CRYSTAL23[12] was used to perform solid-state density functional theory (ss-DFT) simulations on the two crystalline phases of **H2**. The PBE density functional[13] was used with the 6-311G(d,p)[14] basis set. This combination of density functional and basis set has been shown to be reliable for the simulation of molecular crystal structures and dynamics.[15] The PBE functional was augmented with the D3 London dispersion and three-body Axilrod−Teller−Muto repulsion corrections to improve intermolecular noncovalent interaction energies.[16-19] Given the sensitivity of the polymorphs to temperature, geometry optimizations were performed on only the atom positions with the crystallographic lattice parameters fixed at the experimental X-ray values measured at 248 K (Phase I) and 150 K (Phase II). The use of fixed lattice dimension calculations is acceptable given that the measured unit cell volumes change by only about 1% in each phase as a function of temperature. The calculations used a pruned DFT integration grid of 75 radial points and 974 angular points and a sampling of 36 k-points in the irreducible Brillouin zone. Energy convergence thresholds were set at $\Delta E < 10^{-8}$ hartree for geometry optimizations and 10^{-9} hartree for vibrational frequencies.

The agreement between experimental and optimized crystal structure is evidenced in Figures S17 and S18.

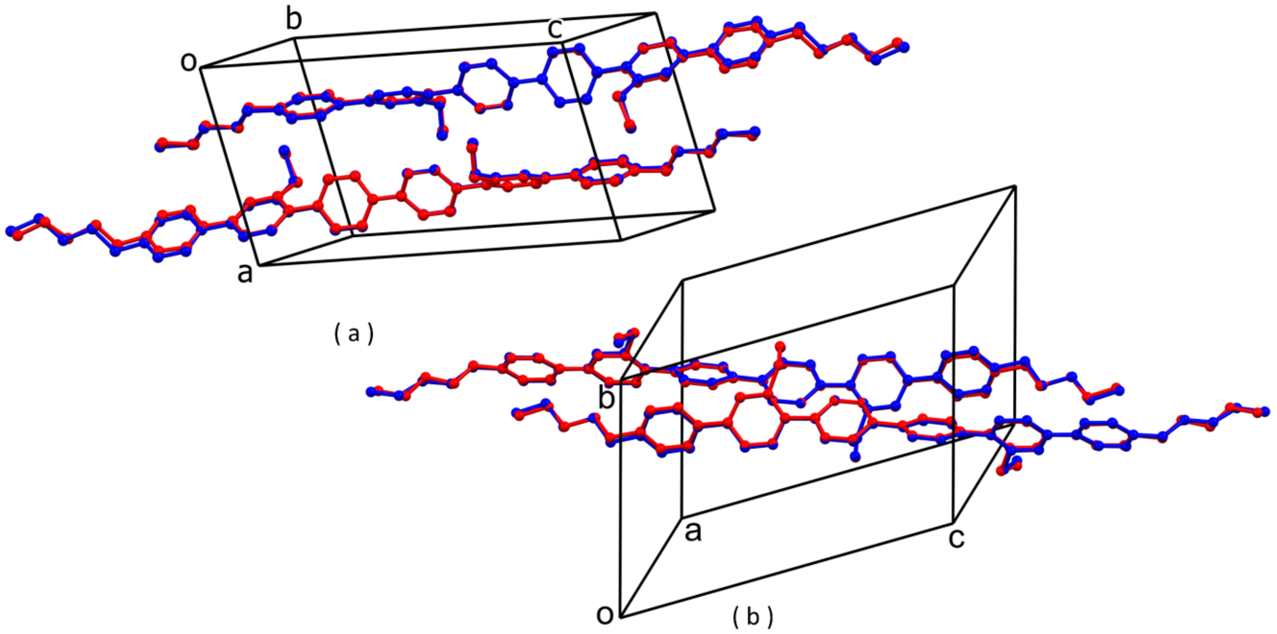

Fig. S17. Comparison between experimental (red) and optimized (blue) phase I at 258 K. (a) and (b) are two different skew views of the unit cell content. H atoms omitted for clarity. Only the major component of the split-disordered ethyl chain is shown.

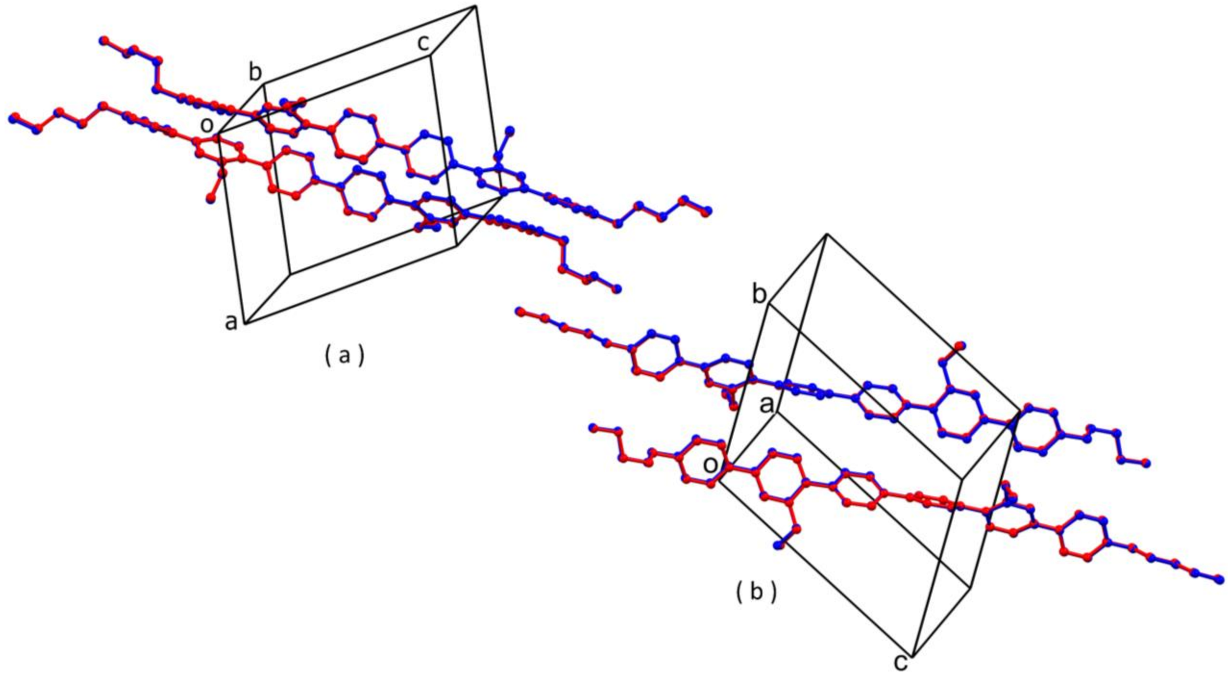

Fig. S18. Comparison between experimental (red) and optimized (blue) phase II at 150 K. (a) and (b) are two different skew views of the unit cell content. H atoms omitted for clarity. Only the major component of the split-disordered pentyl chain is shown.

Normal mode vibrational analyses were based on the numerical derivative of the Hessian matrix calculated with the central-difference formula using two displacements in Cartesian space of each atom in the crystallographic asymmetric unit cell. Computed Raman intensities were determined with the coupled-perturbed Hartree-Fock/Kohn-Sham (CPHF/KS) method,[20-22] with the final values also scaled for experimental laser wavelength and sample temperature. To aid in comparison with experimental observations, the simulated spectra have been convolved with a Lorentzian line shape (empirically determined) having a full-width-at-half-maximum (FWHM) of 8.0 cm^{-1} and 5.0 cm^{-1} for phase I and II, respectively.

All vibrational frequency analyses were performed within the harmonic oscillator limit. This is reasonable since our experimental Raman data show there is only a small amount of frequency shifting as a function of temperature, indicating the vibrations are primarily harmonic in nature. Also, while all experimental vibrations contain some degree of anharmonic character, the harmonic approach has been demonstrated to be adequate for representing the lattice vibrations of a wide array of molecular crystals, including solids with anharmonic properties.[23]

The molecular conformation energy of **H2** was determined by extraction of a single **H2** molecule from the ss-DFT geometry optimized solid and calculating the energy of the isolated molecule. Cohesion energies of crystals were calculated by subtracting the energy of the extracted molecule

(multiplied by the Z of the unit cell, two in this work) from the total solid-state electronic energy of the same unit cell.

7.1. Intramolecular and intermolecular electronic energies

The electronic energies of the geometry-optimized **H2** crystals revealed phase II to be more stable than phase I by 8.52 kJ/mol per molecule. The total electronic energies of the **H2** crystals can be further decomposed into intramolecular conformation and intermolecular cohesion components. The **H2** molecules in phase II were found to have a conformation energy 3.74 kJ/mol per molecule lower than those in phase I. The cohesion energy of phase II was also found to be more favorable, being 4.79 kJ/mol per molecule stronger than phase I. Molecular energies reported in this work have not been corrected for basis set superposition error (BSSE).

The temperature-dependent Gibbs free energy of both phases was calculated and confirmed the monotropic relationship between the polymorphs, with phase II being thermodynamically favored at all temperatures, from 0 K up to the melting temperature (155 °C/428 K), see Fig. S19.

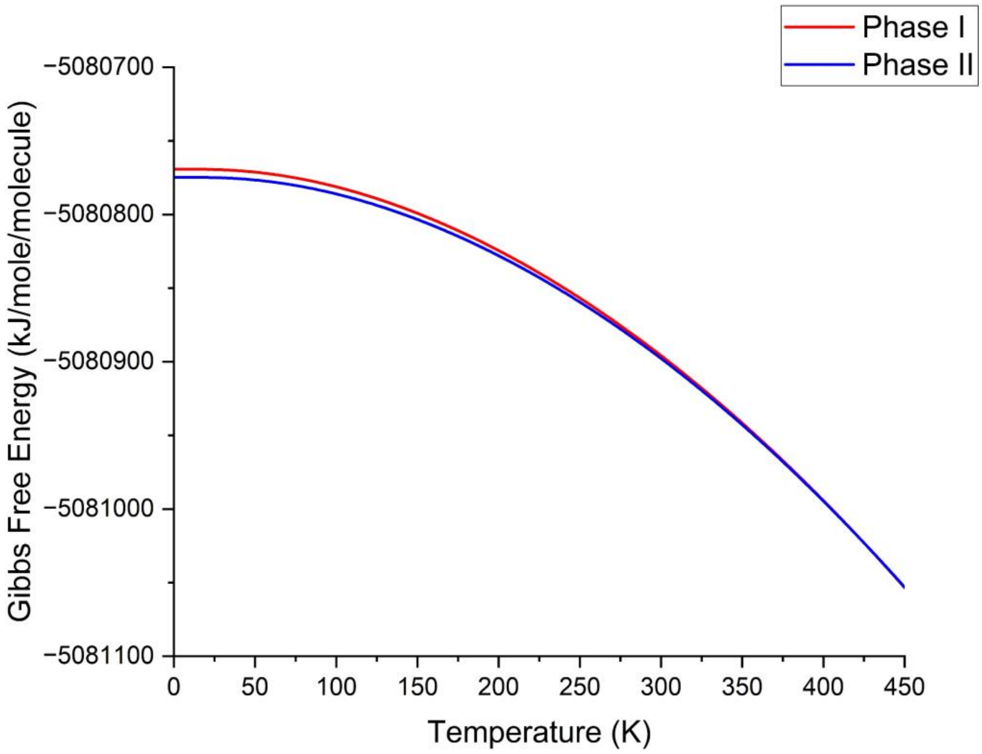

Fig. S19. Calculated temperature-dependent Gibbs free energy of phases I and II of **H2**.

The energy difference at the experimentally identified phase transition temperature (233 K) was 3.01 kJ/mol per molecule, in reasonable agreement with the DSC results. Overall, the electronic and

Gibbs energies clearly indicate that phase I is a metastable crystal form of **H2** that is consistent with the irreversible nature of the phase transition.

7.2 Description of vibrational modes related to phase transition

Calculated frequencies of phases I and II of **H2** are reported in Tables S7-S10. The frequency analyses of both phases yielded no negative (imaginary) vibrational modes, indicating that the geometry-optimized structures of phase I and phase II correspond to minima on the potential energy surfaces. The lowest frequency vibration in phase I of **H2** is a translational motion that has the molecules sliding along the *c*-axis in opposite directions. In a similar way, the lowest frequency vibration in phase II is a translational motion that has the molecules sliding parallel to each other in opposite directions. Movies of the lowest frequency principal vibration of phase I (movie_3) and phase II (movie_4) were produced using *Jmol* program.[24]

Table S7. Description of Raman-active ss-DFT calculated vibrational modes for **H2** phase I below 80 cm^{-1}.

Frequency (cm^{-1})	Major Vibration Motion	Primary Character
21.1032	Translation along the *c*-axis	External
28.3340	Large-amplitude delocalized bending	Internal
36.3623	Libration about the *b*-axis	External
39.3561	Libration about the *a*-axis	External
43.3001	Torsion of terminal chains	Internal
46.9417	Large-amplitude delocalized bending	Internal
50.9247	Libration about the *b*-axis	External
55.0733	Libration about the *a*-axis	External
62.8238	Libration about the *c*-axis	External
64.4539	Torsion of phenyl rings	Internal
65.6166	Large-amplitude delocalized bending	Internal
68.6068	Large-amplitude delocalized bending	Internal
69.2688	Libration about the *a*-axis	External
76.2205	Large-amplitude delocalized bending, phenyl ring torsion	Internal
77.4188	Large-amplitude delocalized bending, phenyl ring torsion	Internal

Table S8. Description of Raman-active ss-DFT calculated vibrational modes for **H2** phase II below 80 cm^{-1}.

Frequency (cm^{-1})	Major Vibration Motion	Primary Character
25.2127	Translation in the *ac*-plane	External
30.6280	Large-amplitude delocalized bending	Internal
36.3692	Libration in the *ac*-plane	External

44.7275	Large-amplitude delocalized bending	Internal
47.1155	Torsion of the terminal chains	Internal
51.2619	Libration in the *ab*-plane	External
53.6864	Libration in the *ac*-plane	External
57.3927	Libration in the *ac*-plane	External
60.3405	Large-amplitude delocalized bending	Internal
63.0314	Libration in the *ac*-plane	External
65.6886	Large-amplitude delocalized bending	Internal
67.8278	Libration in the *ac*-plane	External
70.9747	Large-amplitude delocalized bending	Internal
75.0262	Large-amplitude delocalized bending	Internal
75.5579	Libration in the *ac*-plane	External

Table S9. Calculated vibrational frequencies, for 633 nm incident laser wavelength, for phase I of **H2**. Raman intensities are normalized to a maximum value of 1000.0. IR intensities are provided on a per unit cell basis.

**H2** Phase I PBE/6-311G(d,p)			
Frequency (cm^{-1})	Symmetry	Raman Intensity (arb. units)	IR Intensity (km/mol)
21.1032	Ag	7.96	-
27.2357	Au	-	0.25
28.334	Ag	31.87	-
32.9783	Au	-	0.28
36.3623	Ag	10.84	-
36.7938	Au	-	0.35
39.3561	Ag	14.09	-
41.7849	Au	-	0.59
43.3001	Ag	27.19	-
44.9651	Au	-	0.2
46.9417	Ag	5.61	-
50.9247	Ag	7.77	-
52.3246	Au	-	0.05
55.0733	Ag	11.27	-
55.084	Au	-	0.39
57.675	Au	-	0.26
62.8238	Ag	3.84	-
64.4539	Ag	20.99	-
65.6166	Ag	10.02	-
66.4857	Au	-	0.51
68.6068	Ag	4.91	-
68.6613	Au	-	0.34
69.2688	Ag	13.41	-
72.8783	Au	-	0.95
76.2205	Ag	6.2	-
77.3912	Au	-	0.46
77.4188	Ag	7.44	-
81.582	Au	-	1.14

82.5656	Au	-	1.53
84.0186	Ag	1.1	-
86.6767	Au	-	3.68
87.1785	Au	-	0.14
88.3423	Au	-	1.96
89.1674	Ag	8.44	-
91.7126	Ag	6.26	-
93.8758	Ag	15.72	-
98.4597	Ag	11.73	-
99.0503	Au	-	0.38
101.2724	Ag	6.85	-
101.3499	Au	-	0.44
102.6252	Au	-	0.29
103.723	Ag	3.57	-
104.7259	Ag	4.29	-
104.9616	Au	-	0.83
107.8689	Ag	3.86	-
109.9918	Au	-	1.2
110.8898	Ag	5.85	-
113.8288	Au	-	0.39
114.7037	Ag	7.37	-
115.0776	Au	-	0.07
117.4741	Ag	17.78	-
120.1389	Au	-	0.78
122.1965	Ag	7.19	-
125.1154	Au	-	0.8
126.506	Ag	1.27	-
129.8992	Au	-	2.81
132.5309	Ag	9.41	-
135.5835	Ag	4.73	-
138.4714	Au	-	0.39
142.6313	Ag	0.39	-
143.6576	Au	-	0.95
151.0658	Au	-	3.46
151.2053	Ag	3.82	-
156.2575	Au	-	1.65
156.8888	Ag	1.65	-
159.4705	Au	-	0.07
161.0917	Ag	0.36	-
165.9794	Au	-	0.45
167.0706	Ag	0.82	-
173.2371	Au	-	0.27
174.1441	Ag	0.73	-
178.3985	Au	-	1.01
179.7427	Ag	0.17	-
186.4676	Au	-	2.96

187.5304	Ag	0.45	-
202.9124	Ag	0.65	-
203.308	Au	-	1.15
212.8734	Ag	0.69	-
215.8068	Au	-	1.61
218.1297	Au	-	1.39
218.6064	Ag	0.36	-
221.4306	Ag	0.78	-
222.394	Au	-	1.45
229.8014	Au	-	0.09
233.2786	Ag	0.21	-
234.2132	Ag	1.25	-
236.1601	Au	-	0.72
256.0845	Au	-	0.24
256.8511	Ag	0.27	-
259.9225	Ag	0.23	-
261.0692	Au	-	0.25
278.1979	Au	-	0.25
278.5006	Ag	1.61	-
286.271	Au	-	2.59
286.7213	Ag	1.68	-
293.9659	Au	-	0.96
294.7559	Ag	0.68	-
306.555	Ag	0.94	-
307.0508	Au	-	2.01
321.9525	Au	-	1.85
322.9863	Ag	0.75	-
323.8297	Au	-	0.1
324.9453	Ag	1.75	-
334.3807	Au	-	0.16
334.7286	Ag	2.31	-
338.9481	Au	-	1.04
339.7851	Ag	8.97	-
351.45	Au	-	0.22
353.0126	Ag	0.22	-
359.3868	Au	-	3.23
360.4459	Ag	1.56	-
370.5588	Au	-	1.41
372.2839	Ag	1.8	-
388.8975	Au	-	1.5
389.4275	Ag	5.69	-
393.2395	Au	-	8.72
394.6029	Ag	3.74	-
399.9125	Au	-	0.9
400.2479	Ag	2.16	-
402.4877	Au	-	2.98

405.7058	Ag	14.3	-
407.6503	Au	-	1.4
408.1264	Au	-	0.27
408.5598	Ag	50.38	-
411.0923	Ag	2.52	-
411.2349	Au	-	4.29
413.3803	Ag	24.61	-
416.5941	Au	-	8.25
418.4859	Ag	26.91	-
425.1667	Au	-	13.56
426.3629	Ag	1.07	-
442.9687	Au	-	0.31
443.4646	Ag	0.57	-
445.3495	Ag	8.12	-
447.2589	Au	-	5.01
458.9755	Ag	6.6	-
459.6926	Au	-	2.25
465.9732	Au	-	35.53
466.4784	Ag	21.5	-
476.9588	Au	-	23.38
478.2248	Ag	0.21	-
497.966	Au	-	3.72
498.1361	Ag	0.64	-
512.3194	Au	-	23.74
513.0425	Ag	2.17	-
526.3627	Au	-	31.38
528.6376	Ag	0.4	-
532.4342	Au	-	94.66
534.5681	Ag	0.68	-
542.4574	Ag	2.97	-
542.8414	Au	-	14.66
557.3905	Au	-	29.31
558.3398	Ag	1.22	-
568.2591	Ag	0.83	-
569.5578	Au	-	24.47
580.3399	Au	-	17.11
581.6999	Ag	0.73	-
596.5934	Ag	2.12	-
597.7494	Au	-	13.38
601.7964	Ag	0.64	-
602.0361	Au	-	5.99
614.7159	Au	-	1.43
615.3789	Ag	1.39	-
627.11	Au	-	6.8
627.7892	Ag	1.56	-
629.116	Au	-	6.12

630.2174	Ag	1.04	-
636.2173	Au	-	0.81
637.0399	Ag	0.76	-
637.9055	Ag	1	-
638.0154	Au	-	5.83
639.9339	Au	-	0.01
640.597	Ag	0.25	-
675.4633	Au	-	4.61
675.9464	Ag	0.14	-
690.7716	Ag	1.83	-
691.3447	Au	-	0.6
711.5646	Au	-	3.82
711.9931	Ag	0.53	-
713.0723	Au	-	2.3
713.6232	Ag	0.24	-
714.3617	Au	-	51.65
714.7386	Ag	0.07	-
715.6808	Ag	0.25	-
717.1318	Au	-	2.96
721.439	Ag	1.44	-
721.7063	Au	-	12.38
725.4918	Au	-	3.51
726.3479	Ag	2.17	-
727.3903	Ag	0.37	-
728.3753	Au	-	23.31
737.4489	Ag	0.54	-
737.6898	Au	-	26.7
742.6036	Au	-	11.57
744.1776	Ag	0.57	-
744.6042	Ag	0.08	-
744.6896	Au	-	18.01
749.6801	Au	-	11.94
750.7081	Ag	1.15	-
768.6472	Ag	4.86	-
768.8468	Au	-	15.83
774.5543	Ag	10.02	-
774.8164	Au	-	33.35
781.0975	Au	-	15.21
781.1318	Ag	2.11	-
792.6667	Au	-	111.59
793.1656	Ag	0.34	-
793.465	Au	-	271.74
796.2555	Ag	0.77	-
807.4073	Au	-	249.63
810.8125	Ag	0.43	-
812.1679	Au	-	23.17

812.4907	Au	-	58.73
814.4961	Ag	1.7	-
815.0091	Au	-	63.43
816.1563	Ag	0.32	-
816.6101	Ag	1.95	-
817.7208	Au	-	44.17
818.5216	Ag	0.24	-
820.0496	Au	-	37.48
822.2957	Ag	1.15	-
824.083	Au	-	50.26
826.0337	Ag	0.85	-
827.244	Au	-	33.35
829.265	Ag	2.76	-
830.5202	Au	-	56.07
831.5977	Ag	0.13	-
833.7824	Au	-	7.7
834.2911	Ag	0.11	-
842.4274	Ag	0.18	-
845.1573	Au	-	5.91
852.1457	Ag	0.43	-
853.3153	Au	-	10.58
855.3629	Au	-	7.98
856.7892	Ag	1.88	-
857.4504	Ag	6.42	-
858.7345	Au	-	6.45
868.0549	Ag	0.4	-
868.0884	Au	-	52.09
875.619	Au	-	27.17
876.1768	Ag	0.13	-
890.7977	Ag	1.49	-
891.0961	Au	-	5.21
891.2537	Au	-	8.87
891.6093	Ag	1.56	-
924.9655	Ag	0.12	-
925.1777	Au	-	10.73
928.6144	Au	-	1.25
930.7928	Ag	0.06	-
934.3549	Au	-	0.43
938.1015	Au	-	2.18
938.3515	Ag	0.1	-
939.2368	Ag	0.49	-
940.5875	Ag	0.17	-
940.6603	Au	-	3.27
941.3272	Au	-	0.66
943.3483	Ag	0.33	-
944.2784	Ag	0.19	-

944.4085	Au	-	7.07
945.7043	Au	-	8.27
947.2666	Ag	0.05	-
948.8861	Ag	0.48	-
949.4481	Au	-	8.56
951.5852	Au	-	6.73
955.736	Ag	2.04	-
957.9476	Au	-	4.65
957.982	Ag	0.24	-
959.3748	Ag	0.41	-
959.7755	Au	-	14.16
967.991	Au	-	10.05
968.4392	Ag	0.43	-
975.4206	Au	-	13.34
977.2228	Ag	0.33	-
986.2944	Au	-	128.38
986.4648	Ag	0.18	-
991.9032	Au	-	1.23
992.8323	Ag	0.6	-
998.8614	Ag	0.22	-
999.8006	Au	-	0.29
999.9206	Au	-	0.13
1000.395	Ag	0.13	-
1000.722	Au	-	4.77
1000.813	Ag	0.3	-
1005.385	Ag	2.03	-
1005.861	Au	-	11.07
1006.477	Ag	0.07	-
1007.114	Au	-	9.84
1013.265	Ag	0.4	-
1014.032	Au	-	1.24
1024.464	Ag	0.53	-
1025.441	Au	-	4.38
1026.423	Au	-	2.76
1026.477	Ag	0.28	-
1028.525	Au	-	4.18
1028.814	Ag	0.35	-
1034.517	Au	-	5.22
1035.196	Ag	0.21	-
1037.366	Au	-	6.38
1037.946	Ag	0.19	-
1049.091	Au	-	19.27
1049.174	Ag	0.5	-
1055.081	Au	-	0.55
1055.332	Ag	0.77	-
1055.651	Au	-	1.49

1055.783	Ag	0.16	-
1062.612	Ag	1	-
1062.666	Au	-	0.01
1063.9	Ag	0.45	-
1064.618	Au	-	5.54
1065.008	Ag	0.3	-
1065.391	Au	-	2.55
1069.261	Ag	0.05	-
1070.272	Au	-	7.35
1071.118	Au	-	15.22
1071.244	Ag	0.02	-
1098.914	Au	-	29.44
1099.034	Ag	1.12	-
1099.515	Au	-	14.8
1099.689	Ag	2.55	-
1105.305	Ag	0.09	-
1105.324	Au	-	10.69
1113.492	Ag	3.22	-
1113.546	Au	-	52.01
1115.853	Au	-	20.77
1115.9	Ag	0.3	-
1117.795	Au	-	33.33
1118.301	Ag	2.04	-
1127.677	Au	-	22.53
1127.834	Ag	7.74	-
1129.433	Ag	8.21	-
1129.785	Au	-	16.34
1169.935	Ag	16.26	-
1169.985	Au	-	12.16
1170.386	Au	-	2.3
1171.026	Ag	6.64	-
1171.946	Au	-	11
1172.614	Ag	5.05	-
1179.359	Au	-	24.01
1179.479	Ag	2.92	-
1181.564	Ag	0.92	-
1182.203	Au	-	19.92
1182.864	Au	-	3.9
1183.223	Ag	116.64	-
1184.46	Ag	2.55	-
1185.387	Au	-	4.55
1185.897	Ag	12.58	-
1186.572	Au	-	7.68
1203.081	Au	-	6.25
1203.153	Ag	0.16	-
1204.92	Ag	0.67	-

1206.418	Au	-	4.23
1217.688	Ag	2.02	-
1217.716	Au	-	1.5
1219.075	Ag	4.08	-
1219.46	Au	-	0.47
1227.581	Au	-	0.79
1227.734	Ag	3.33	-
1245.332	Ag	61.68	-
1245.536	Au	-	0.85
1247.601	Ag	18.37	-
1247.794	Au	-	5.36
1249.998	Au	-	5.26
1250.102	Ag	1.3	-
1252.569	Ag	35.4	-
1253.172	Au	-	12.68
1259.074	Ag	20.38	-
1259.152	Au	-	9
1267.19	Au	-	24.59
1267.567	Ag	9.95	-
1273.012	Ag	17.4	-
1273.037	Au	-	1.48
1276.758	Au	-	2.82
1276.983	Ag	3.82	-
1277.184	Au	-	8.52
1278.283	Au	-	3.7
1278.325	Ag	0.68	-
1279.407	Ag	0.71	-
1280.285	Ag	23.78	-
1280.713	Au	-	2.39
1281.367	Ag	22.5	-
1282.558	Au	-	6.22
1283.642	Au	-	5.71
1283.7	Ag	52.92	-
1284.529	Au	-	7.19
1285.481	Ag	2.11	-
1286.149	Ag	10.98	-
1286.261	Au	-	3.19
1286.989	Au	-	0.23
1287.726	Ag	0.21	-
1287.965	Ag	5.15	-
1288.173	Au	-	8.49
1289.244	Ag	1.9	-
1289.289	Au	-	3.03
1290.727	Au	-	21.29
1291.332	Ag	110.9	-
1293.592	Ag	114.28	-

1293.647	Au	-	12.49
1300.044	Au	-	2.44
1300.465	Ag	39.34	-
1302.668	Ag	14.48	-
1303.503	Au	-	28.66
1313.982	Ag	4.3	-
1314.474	Au	-	11.1
1322.584	Ag	0.47	-
1323.474	Au	-	1.98
1324.804	Au	-	4.23
1325.635	Ag	10.98	-
1330.14	Au	-	6.53
1330.877	Ag	0.35	-
1336.618	Au	-	3.86
1337.074	Ag	1.21	-
1337.912	Au	-	0.4
1337.961	Ag	6.85	-
1344.975	Au	-	3.69
1345.039	Ag	3.29	-
1345.656	Ag	0.12	-
1345.94	Au	-	5.62
1346.065	Ag	0.19	-
1346.537	Au	-	2.02
1346.814	Au	-	4.13
1346.84	Ag	1.44	-
1354.724	Ag	0.1	-
1355.615	Au	-	12.87
1356.355	Au	-	9.48
1357.244	Ag	0.42	-
1358.338	Au	-	14.71
1359.477	Ag	0.8	-
1361.512	Ag	0.15	-
1361.632	Au	-	13.63
1391.966	Ag	2.77	-
1392.129	Au	-	28.83
1394.363	Ag	4.71	-
1394.811	Au	-	30.69
1396.898	Au	-	44.78
1397.92	Ag	0.51	-
1401.172	Ag	0.06	-
1401.587	Au	-	4.32
1412.52	Ag	0.81	-
1412.524	Au	-	5.14
1415.776	Au	-	11.68
1416.856	Ag	0.23	-
1424.984	Au	-	58.21

1425.555	Ag	0.39	-
1429.71	Au	-	5.37
1430.328	Ag	1.87	-
1431.006	Au	-	1.79
1431.477	Ag	0.32	-
1434.756	Au	-	5.51
1434.921	Ag	0.44	-
1435.602	Au	-	18.3
1435.887	Ag	0.39	-
1437.292	Au	-	6.86
1437.455	Ag	0.72	-
1437.512	Au	-	10.13
1438.466	Ag	1	-
1439.438	Ag	0.76	-
1439.638	Au	-	58.36
1440.834	Au	-	8.21
1441.337	Ag	0.85	-
1443.089	Au	-	56.51
1445.129	Ag	0.51	-
1445.479	Au	-	20.45
1446.326	Ag	0.27	-
1447.252	Au	-	38.04
1448.576	Ag	0.44	-
1451.287	Au	-	9.03
1451.9	Au	-	6.3
1451.921	Ag	0.46	-
1452.604	Ag	0.13	-
1454.337	Au	-	21.47
1455.668	Ag	0.12	-
1458.891	Au	-	57.13
1459.571	Ag	0.3	-
1460.538	Ag	0.43	-
1460.573	Au	-	17.12
1462.391	Au	-	107.81
1462.439	Ag	0.45	-
1467.894	Ag	0.28	-
1469.004	Au	-	729.06
1473.225	Au	-	26.21
1474.514	Ag	39.99	-
1486.576	Au	-	13.9
1487.907	Ag	1.12	-
1503.416	Ag	4.93	-
1505.35	Au	-	7.09
1508.44	Au	-	59.56
1509.286	Ag	0.18	-
1513.72	Ag	0.24	-

1514.17	Au	-	2.71
1525.01	Ag	6.28	-
1525.294	Au	-	5.44
1536.882	Au	-	28.97
1536.993	Ag	0.41	-
1547.1	Au	-	5.98
1547.215	Ag	14.8	-
1559.347	Ag	0.74	-
1559.579	Au	-	6.26
1564.75	Ag	14.3	-
1564.93	Au	-	1.1
1566.759	Ag	7.27	-
1566.932	Au	-	3.02
1591.018	Ag	1000	-
1591.969	Au	-	38.55
1595.739	Au	-	48.61
1595.977	Ag	32.6	-
1601.228	Ag	2.01	-
1601.952	Au	-	0.51
1607.807	Au	-	4.23
1609.135	Ag	0.5	-
1609.938	Ag	14.73	-
1610.539	Au	-	1.08
1612.188	Ag	4.37	-
1612.96	Au	-	6.63
2925.032	Ag	2.22	-
2925.112	Au	-	46.16
2935.33	Au	-	23.87
2936.066	Ag	2.46	-
2940.609	Ag	0.75	-
2940.615	Au	-	97.15
2942.81	Au	-	10.62
2943.061	Ag	4.97	-
2947.926	Au	-	64.56
2947.993	Ag	0.4	-
2950.666	Ag	2.73	-
2950.734	Au	-	50.53
2955.599	Ag	1.68	-
2955.708	Au	-	178.41
2956.061	Au	-	72.36
2956.127	Ag	3.09	-
2958.331	Au	-	9.86
2958.438	Ag	5.1	-
2959.68	Au	-	113.36
2961.006	Ag	0.54	-
2962.041	Au	-	133.86

2962.618	Au	-	279.65
2962.693	Ag	3.75	-
2962.728	Ag	1.6	-
2965.986	Ag	2.12	-
2966.359	Au	-	224.72
2967.163	Au	-	38.55
2967.175	Ag	3.8	-
2970.552	Au	-	117.37
2970.566	Ag	1.36	-
2974.346	Au	-	50.86
2974.568	Ag	1.9	-
2987.779	Au	-	15.46
2987.792	Ag	0.19	-
2988.233	Au	-	9.21
2988.322	Ag	0.55	-
2994.661	Au	-	93.34
2994.686	Ag	1.86	-
2998.326	Ag	1.53	-
2998.34	Au	-	5.59
2998.713	Ag	1.33	-
2998.926	Au	-	57.72
3010.84	Ag	0.19	-
3011.079	Au	-	216.27
3013.278	Au	-	128.66
3013.42	Ag	0.37	-
3026.385	Au	-	6.72
3026.434	Ag	1.27	-
3029.617	Au	-	171.15
3030.756	Ag	0.61	-
3032.992	Ag	0.54	-
3033.091	Au	-	213.67
3038.323	Ag	0.3	-
3038.341	Au	-	100.63
3039.57	Au	-	106.34
3040.38	Ag	0.76	-
3041.148	Au	-	157.97
3041.359	Ag	0.6	-
3041.823	Au	-	66.53
3042.717	Ag	0.84	-
3050.695	Ag	0.63	-
3050.776	Au	-	97.77
3052.968	Au	-	102.98
3053.037	Ag	0.28	-
3097.146	Ag	1.24	-
3097.531	Au	-	49.6
3098.905	Ag	0.97	-

3099.313	Au	-	23.49
3102.978	Ag	0.72	-
3103.409	Au	-	56.13
3105.503	Ag	0.35	-
3105.746	Au	-	18.61
3107.53	Ag	1.51	-
3107.575	Au	-	21.54
3108.798	Au	-	6.34
3108.817	Ag	0.17	-
3112.189	Au	-	10.26
3112.475	Ag	0.53	-
3113.061	Au	-	37.2
3113.398	Ag	0.2	-
3113.4	Au	-	15.77
3113.779	Ag	0.55	-
3114.316	Ag	0.55	-
3114.609	Au	-	16.57
3115.909	Au	-	2.47
3116.046	Ag	0.27	-
3117.129	Ag	1.2	-
3117.35	Au	-	23.35
3120.284	Ag	0.83	-
3120.419	Au	-	15.34
3121.144	Ag	1.53	-
3121.163	Au	-	16.98
3122.417	Au	-	4.38
3122.822	Ag	0.18	-
3126.718	Au	-	8.72
3127.749	Ag	0.34	-
3130.671	Ag	1.03	-
3130.711	Au	-	6.16
3132.385	Ag	2.65	-
3132.852	Au	-	2.44
3133.725	Au	-	9.17
3134.231	Ag	0.98	-
3135.977	Au	-	10.8
3136.168	Ag	0.75	-
3136.641	Au	-	22.85
3136.793	Au	-	14.18
3137.745	Ag	1.14	-
3139.884	Ag	6.92	-

Table S10. Calculated vibrational frequencies, for 633 nm incident laser wavelength, for phase II of **H2**. Raman intensities are normalized to a maximum value of 1000.0. IR intensities are provided on a per unit cell basis.

**H2** Phase II PBE/6-311G(d,p)			
Frequency (cm^{-1})	Symmetry	Raman Intensity (arb. units)	IR Intensity (Km/mol)
25.2127	Ag	9.9	-
30.628	Ag	17.6	-
34.1426	Au	-	0.54
36.3692	Ag	68.4	-
38.6598	Au	-	0.04
42.0308	Au	-	0.09
44.5814	Au	-	0.97
44.7275	Ag	10.8	-
47.1155	Ag	11.34	-
47.7096	Au	-	0.45
51.2619	Ag	7.53	-
53.6864	Ag	48.64	-
55.058	Au	-	0.48
57.3927	Ag	24.15	-
60.3405	Ag	7.37	-
60.4545	Au	-	0.16
63.0314	Ag	11.37	-
65.6886	Ag	9.47	-
65.7536	Au	-	0.12
67.8278	Ag	10.51	-
70.109	Au	-	0.26
70.7533	Au	-	0.7
70.9747	Ag	4.08	-
75.0262	Ag	6.07	-
75.2133	Au	-	0.51
75.5579	Ag	2.86	-
77.7242	Au	-	1
78.116	Au	-	1.19
82.0219	Ag	2.85	-
86.1392	Ag	13.05	-
86.8908	Au	-	1.19
87.3	Ag	4.14	-
87.7558	Au	-	0.18
88.3831	Ag	1.81	-
91.0818	Au	-	3.41
94.871	Ag	9.95	-
94.9923	Au	-	0.42
97.3741	Ag	2.51	-
99.0429	Au	-	0.42
101.9464	Au	-	0.23
103.2091	Ag	2.06	-
104.4463	Au	-	0.24
106.4269	Ag	10.76	-
107.2822	Au	-	1.74

110.6624	Au	-	1.54
114.2968	Au	-	1.92
114.4428	Ag	3.03	-
116.5417	Ag	9.24	-
120.6259	Ag	9.07	-
124.0779	Au	-	0.61
124.2042	Au	-	0.34
127.461	Ag	6.89	-
128.6786	Au	-	0.28
131.3781	Ag	2.05	-
134.1479	Au	-	0.78
135.8386	Ag	2.14	-
138.5856	Au	-	0.23
139.5926	Ag	8.21	-
143.8607	Ag	8.07	-
144.3714	Au	-	1.32
149.5736	Ag	2.64	-
150.5906	Au	-	2.21
154.1474	Ag	4.83	-
158.0769	Ag	2.47	-
158.9566	Au	-	1.03
166.3118	Au	-	2.21
167.5168	Ag	1.04	-
170.0092	Au	-	2.03
172.1564	Ag	1.01	-
174.7885	Ag	1.65	-
175.5929	Au	-	1.9
190.0834	Au	-	3.07
192.7346	Ag	0.39	-
209.9392	Ag	0.36	-
210.7651	Au	-	1.39
218.2657	Au	-	0.8
219.8452	Ag	1.29	-
223.9785	Au	-	0.6
227.499	Ag	0.87	-
230.3662	Ag	0.37	-
231.0644	Au	-	1.62
238.0633	Au	-	0.95
240.1516	Ag	0.32	-
242.4004	Ag	1.1	-
242.6571	Au	-	0.39
243.8649	Au	-	1.83
243.9402	Ag	0.17	-
259.7406	Au	-	0.2
260.185	Ag	0.49	-
269.5388	Ag	0.34	-

270.2032	Au	-	0.37
273.7151	Au	-	1.22
274.2785	Ag	0.88	-
283.1339	Au	-	0.05
285.7745	Ag	0.98	-
285.7986	Au	-	2
286.5415	Ag	2.74	-
300.0137	Au	-	1.24
300.4511	Ag	0.44	-
321.7527	Au	-	1.05
322.3605	Ag	0.44	-
331.0193	Ag	1.1	-
333.1471	Au	-	2.89
335.2343	Au	-	0.51
335.4491	Ag	2.42	-
337.8284	Au	-	0.78
338.561	Ag	0.91	-
344.2789	Ag	10.65	-
344.6422	Au	-	0.23
362.0436	Au	-	5.2
364.3095	Ag	1.21	-
373.7309	Au	-	2.62
374.0994	Ag	1.85	-
391.8201	Au	-	2.11
392.6979	Ag	1.91	-
397.1603	Ag	0.75	-
398.5755	Au	-	7.55
401.1124	Ag	4.36	-
401.144	Au	-	4.46
404.0751	Au	-	2.38
408.4128	Ag	18.28	-
408.9278	Ag	45.96	-
409.6036	Au	-	4.35
411.6415	Au	-	4.11
411.7693	Ag	9.98	-
413.6119	Au	-	0.31
416.01	Ag	16.92	-
416.3089	Au	-	4.82
423.2505	Ag	56.01	-
428.993	Au	-	10.9
429.9668	Ag	2.04	-
444.1	Ag	1.22	-
444.3683	Au	-	5.25
458.1356	Au	-	5.01
458.369	Ag	0.41	-
461.5793	Ag	26.41	-

462.1561	Au	-	2.91
474.1952	Au	-	46.09
474.235	Ag	7.6	-
478.9147	Au	-	21.94
479.3341	Ag	0.47	-
499.8244	Au	-	0.97
499.8324	Ag	0.85	-
512.7061	Au	-	24.52
513.7153	Ag	2.12	-
531.8238	Au	-	60.27
531.9957	Ag	0.17	-
537.6501	Ag	0.1	-
538.2947	Au	-	71.94
546.8979	Ag	2.36	-
546.9793	Au	-	17.08
559.0197	Au	-	14.85
559.3715	Ag	2.51	-
568.142	Ag	0.33	-
568.8796	Au	-	39.39
584.5973	Au	-	27.21
585.6519	Ag	1.33	-
600.0136	Au	-	6.61
600.5367	Ag	0.47	-
607.2085	Au	-	9.9
607.8541	Ag	3.09	-
614.9891	Ag	1.54	-
615.6511	Au	-	0.78
626.7541	Au	-	9.72
627.3785	Ag	2.55	-
628.3329	Au	-	8.21
628.632	Ag	1.48	-
635.6869	Au	-	1.04
635.959	Ag	0.06	-
637.1694	Au	-	4.06
637.3741	Ag	1.55	-
640.1347	Au	-	0.97
640.4991	Ag	0.47	-
675.4045	Au	-	6.92
675.9961	Ag	0.13	-
692.8768	Ag	2.58	-
692.9136	Au	-	0.93
708.8779	Ag	0.08	-
709.7352	Au	-	42.57
713.1975	Au	-	2.04
713.2005	Ag	0.37	-
715.9578	Ag	0.47	-

716.1162	Au	-	16.19
717.0352	Au	-	7.58
719.1317	Ag	0.13	-
724.8682	Ag	0.22	-
725.0859	Au	-	2.27
728.0517	Ag	4.02	-
728.6853	Au	-	23.45
733.3142	Au	-	7.55
734.253	Ag	0.42	-
737.8255	Au	-	22.35
738.0819	Ag	0.43	-
740.1876	Ag	0.17	-
740.4783	Au	-	9.62
742.5827	Au	-	15.88
744.5846	Ag	0.31	-
750.3667	Au	-	13.76
752.2994	Ag	1.48	-
770.3046	Ag	10.22	-
770.442	Au	-	13.47
774.9562	Ag	7.47	-
775.9832	Au	-	54.48
781.5791	Au	-	14.39
781.6667	Ag	4.61	-
793.8008	Au	-	327.29
794.4484	Ag	0.35	-
796.0181	Au	-	64.41
797.8296	Ag	1.78	-
809.6778	Au	-	296.3
813.2192	Ag	1.83	-
813.2261	Au	-	24.06
814.9129	Ag	2.12	-
815.3359	Au	-	154.12
817.3751	Ag	1.56	-
820.61	Ag	0.15	-
820.8062	Au	-	50.09
822.1135	Ag	0.71	-
823.2668	Au	-	8.89
826.5892	Au	-	8.13
827.1965	Ag	0.23	-
829.2621	Au	-	34.63
830.8123	Ag	0.22	-
830.9268	Au	-	46.04
833.4025	Au	-	15.63
833.4911	Ag	0.57	-
835.4166	Au	-	25.9
835.6117	Ag	0.21	-

836.7544	Ag	0.8	-
841.7688	Au	-	8.14
841.9617	Ag	0.52	-
854.0391	Au	-	34.26
856.1293	Ag	0.14	-
856.6292	Ag	1.47	-
858.1585	Au	-	5.19
860.1268	Au	-	4.83
860.4698	Ag	3.11	-
863.3811	Ag	5.9	-
864.7979	Au	-	21.93
872.2764	Au	-	24.71
872.4195	Ag	0.18	-
876.8947	Au	-	19.12
877.2153	Ag	0.32	-
897.0002	Au	-	4.07
897.2009	Ag	1.41	-
925.6852	Au	-	0.86
925.8799	Ag	0.05	-
931.5569	Ag	0.01	-
931.7025	Au	-	1.5
937.6309	Ag	0.18	-
939.2662	Ag	0.17	-
940.273	Au	-	1.57
941.1488	Au	-	0.06
942.3615	Au	-	2.13
942.8131	Ag	0.07	-
943.9729	Au	-	0.86
944.2085	Au	-	1.62
944.7568	Ag	0.06	-
946.9024	Au	-	3.67
947.1022	Ag	0.03	-
948.5296	Ag	1.8	-
949.7167	Au	-	1.71
953.3573	Ag	0.78	-
954.3553	Au	-	9.7
954.5329	Ag	0.43	-
958.1685	Au	-	14.65
958.6386	Ag	1.09	-
958.8741	Au	-	3.59
959.5958	Au	-	15.16
962.1521	Ag	0.35	-
962.7659	Ag	0.73	-
968.9006	Au	-	11.49
969.5688	Ag	0.58	-
975.6956	Au	-	14.05

977.1985	Ag	0.58	-
987.0222	Au	-	113.98
987.5549	Ag	0.27	-
994.4875	Ag	0.54	-
994.9774	Au	-	4.67
1001.283	Au	-	1.02
1001.395	Ag	0.36	-
1001.881	Au	-	9.13
1002.046	Ag	0.03	-
1005.487	Au	-	3.54
1005.97	Ag	1.52	-
1007.597	Au	-	16.73
1007.904	Ag	0.86	-
1015.447	Ag	0.72	-
1015.684	Au	-	2.41
1026.452	Au	-	2
1027.866	Ag	0.3	-
1028.026	Au	-	7.6
1028.812	Ag	0.21	-
1030.889	Au	-	8.01
1031.503	Ag	0.54	-
1034.863	Ag	0.29	-
1035.402	Au	-	3.35
1042.088	Ag	0.13	-
1042.213	Au	-	6.53
1049.868	Au	-	13.49
1049.965	Ag	0.6	-
1059.427	Ag	1.14	-
1059.801	Au	-	2.96
1059.944	Ag	0.29	-
1062.501	Au	-	2.19
1063.729	Ag	0.29	-
1064.5	Au	-	2.4
1064.991	Au	-	5.5
1065.02	Ag	0.61	-
1067.603	Ag	0.23	-
1067.925	Au	-	0.25
1068.302	Ag	0.21	-
1068.948	Au	-	7.28
1074.112	Ag	0.14	-
1074.991	Au	-	11.44
1096.248	Ag	2.6	-
1097.155	Au	-	42
1102.264	Au	-	29.13
1102.382	Ag	1.64	-
1106.891	Ag	0.05	-

1108.501	Au	-	12.97
1110.788	Ag	1.04	-
1111.507	Au	-	47.28
1114.924	Ag	0.09	-
1115.159	Au	-	13.27
1116.356	Ag	1.38	-
1117.175	Au	-	11.51
1125.12	Ag	9.02	-
1126.036	Au	-	19.55
1127.026	Au	-	27.38
1129.068	Ag	9.12	-
1166.863	Au	-	17.38
1166.906	Ag	3.27	-
1168.092	Ag	2	-
1168.152	Au	-	6.31
1170.809	Au	-	0.69
1171.142	Ag	25.8	-
1173.399	Ag	0.36	-
1174.056	Au	-	10.96
1178.424	Ag	17.18	-
1178.581	Au	-	24.68
1180.74	Au	-	4.37
1180.93	Ag	1.59	-
1182.647	Ag	6.49	-
1183.657	Au	-	16.78
1185.206	Ag	91.86	-
1185.655	Au	-	4.02
1205.755	Ag	1.89	-
1206.164	Au	-	4.09
1207.035	Ag	0.19	-
1207.046	Au	-	0.64
1217.395	Au	-	2.3
1218.275	Ag	4.62	-
1219.209	Ag	0.61	-
1220.918	Au	-	5.08
1229.785	Au	-	3.22
1229.832	Ag	3.95	-
1241.117	Au	-	3.56
1241.497	Ag	29.28	-
1247.024	Au	-	2.77
1247.734	Ag	0.99	-
1254.43	Au	-	10.95
1254.77	Ag	27.25	-
1255.199	Ag	41.69	-
1255.988	Au	-	6.94
1257.221	Au	-	5.46

1260.052	Ag	3.71	-
1265.676	Ag	0.05	-
1267.108	Au	-	2.53
1271.039	Au	-	22.08
1271.912	Ag	24.07	-
1274.353	Au	-	7.95
1275.162	Ag	5.11	-
1275.293	Ag	5.51	-
1276.005	Au	-	1.07
1279.937	Au	-	9.02
1280.375	Ag	18.49	-
1281.919	Ag	4.37	-
1282.142	Au	-	4.8
1284.242	Au	-	6.75
1284.392	Ag	3.63	-
1284.739	Au	-	2.37
1284.9	Ag	34.44	-
1285.398	Ag	0.93	-
1285.797	Au	-	9.03
1287.74	Au	-	2.7
1288.467	Ag	4.05	-
1288.94	Au	-	0.88
1289.504	Ag	13.54	-
1290.366	Ag	5.62	-
1291.666	Au	-	7.94
1291.941	Au	-	6.13
1293.251	Ag	5.44	-
1294.509	Au	-	0.45
1295.413	Ag	239.98	-
1298.537	Au	-	6.21
1300.192	Ag	32.29	-
1301.625	Au	-	5.81
1301.766	Ag	47.39	-
1304.424	Au	-	30.33
1304.927	Ag	32.67	-
1316.069	Ag	10.19	-
1316.708	Au	-	13.21
1326.418	Au	-	9.99
1326.618	Ag	6.13	-
1330.506	Ag	0.85	-
1330.867	Au	-	11.94
1331.399	Au	-	2.16
1332.503	Ag	2.71	-
1336.862	Ag	4.24	-
1337.023	Au	-	0.54
1339.531	Au	-	1.02

1339.544	Ag	1.93	-
1342.879	Au	-	4.27
1343.136	Ag	1.81	-
1346.845	Ag	2.72	-
1347.237	Au	-	1.35
1347.514	Au	-	2.34
1348.021	Ag	2.65	-
1348.849	Ag	0.13	-
1349.34	Au	-	8.15
1355.791	Au	-	4.97
1356.095	Ag	0.04	-
1357.087	Ag	0.55	-
1357.644	Au	-	23.84
1358.943	Au	-	12.87
1359.969	Ag	1.08	-
1363.4	Au	-	13.81
1364.238	Ag	0.12	-
1395.013	Au	-	45.88
1395.641	Ag	0.65	-
1395.811	Au	-	3.38
1397.141	Ag	5.11	-
1399.308	Au	-	42.42
1400.281	Ag	1.22	-
1403.198	Ag	0.14	-
1404.466	Au	-	9.03
1414.573	Ag	0.76	-
1414.831	Au	-	11.7
1418.141	Au	-	8.17
1418.568	Ag	0.07	-
1421.651	Ag	0.68	-
1421.951	Au	-	28.09
1424.042	Ag	0.99	-
1424.302	Au	-	17.49
1430.134	Au	-	31.3
1431.575	Au	-	28.74
1431.634	Ag	0.41	-
1432.049	Ag	0.46	-
1433.978	Ag	1.32	-
1434.187	Au	-	2.23
1437.265	Ag	0.86	-
1437.745	Au	-	15.39
1438.429	Au	-	2.41
1438.457	Ag	1.02	-
1439.624	Au	-	25.57
1441.743	Ag	0.84	-
1443.07	Au	-	13.38

1443.51	Ag	0.26	-
1445.71	Au	-	61.45
1446.028	Ag	0.26	-
1447.133	Ag	0.21	-
1447.537	Au	-	16.46
1448.425	Au	-	31.67
1449.222	Ag	0.28	-
1449.684	Au	-	2.95
1449.917	Ag	0.32	-
1453.492	Au	-	58.42
1453.855	Ag	0.25	-
1454.572	Au	-	26.48
1455.022	Ag	0.27	-
1456.832	Ag	0.1	-
1457.007	Au	-	43.66
1458.597	Ag	0.24	-
1459.377	Au	-	56.92
1463.253	Au	-	13.19
1464.236	Ag	0.58	-
1469.768	Ag	0.02	-
1470.708	Au	-	682.3
1474.851	Au	-	21.07
1476.367	Ag	41.36	-
1488.684	Au	-	13.01
1489.027	Ag	0.84	-
1504.981	Ag	4.64	-
1505.181	Au	-	0.63
1510.505	Ag	0.62	-
1510.679	Au	-	62.89
1515.31	Ag	0.28	-
1515.407	Au	-	4.33
1527.596	Ag	5.7	-
1528.078	Au	-	2.08
1539.72	Au	-	28.04
1540.027	Ag	0.5	-
1549.141	Ag	15.27	-
1549.292	Au	-	5.19
1560.701	Au	-	2.85
1561.026	Ag	0.83	-
1567.186	Ag	12.93	-
1567.516	Au	-	1.21
1568.937	Ag	7.71	-
1569.541	Au	-	3.52
1595.114	Ag	1000	-
1596.461	Au	-	2.6
1599.252	Au	-	72.25

1599.472	Ag	66.63	-
1604.261	Ag	2.77	-
1604.46	Au	-	1.22
1610.479	Au	-	2.7
1610.509	Ag	2.14	-
1612.328	Ag	9.51	-
1612.36	Au	-	5.97
1613.434	Au	-	5.94
1614.437	Ag	2.04	-
2936.052	Au	-	55.6
2936.173	Ag	2.02	-
2937.07	Au	-	142.82
2937.225	Ag	3.16	-
2942.037	Au	-	62.15
2942.057	Ag	3.96	-
2948.4	Au	-	90.26
2948.612	Ag	3.42	-
2952.503	Au	-	43.51
2952.589	Ag	2.25	-
2956.2	Ag	1.04	-
2956.514	Au	-	44.93
2959.864	Au	-	150.88
2960.549	Ag	0.98	-
2960.7	Au	-	25.18
2962.704	Ag	4.16	-
2963.275	Au	-	193.57
2963.742	Ag	2.92	-
2964.109	Au	-	30.5
2964.831	Ag	8.99	-
2965.743	Ag	0.56	-
2966.355	Au	-	300.71
2967.826	Au	-	19.54
2968.328	Ag	1.91	-
2970.478	Au	-	70.34
2970.517	Ag	1.53	-
2972.037	Au	-	100.66
2972.583	Ag	2.7	-
2976.264	Ag	1.45	-
2976.545	Au	-	107.59
2976.874	Au	-	290.13
2977.025	Ag	0.66	-
2988.621	Ag	0.68	-
2988.63	Au	-	1.57
2997.468	Au	-	81.18
2997.537	Ag	0.71	-
3000.775	Ag	1.72	-

3000.822	Au	-	48.46
3001.663	Au	-	33.36
3001.723	Ag	1.06	-
3017.351	Ag	0.5	-
3017.394	Au	-	206.52
3019.006	Au	-	4.21
3019.179	Ag	1.83	-
3021.203	Au	-	18.92
3021.285	Ag	1.47	-
3022.624	Ag	0.15	-
3023.232	Au	-	93.21
3030.243	Au	-	248.57
3031.049	Ag	0.61	-
3032.357	Au	-	223.65
3033.974	Ag	0.64	-
3035.734	Au	-	51.41
3036.535	Ag	1.09	-
3044.038	Au	-	180.05
3044.137	Ag	0.19	-
3046.187	Ag	0.3	-
3046.238	Au	-	15.7
3048.592	Ag	0.89	-
3048.847	Au	-	68.77
3054.069	Ag	0.71	-
3054.177	Au	-	54.5
3054.256	Ag	1.34	-
3054.339	Au	-	66.04
3091.72	Au	-	33.31
3091.788	Ag	2.08	-
3093.56	Au	-	42.64
3093.591	Ag	1.33	-
3096.169	Au	-	60.71
3096.353	Ag	1.12	-
3102.418	Au	-	41.38
3102.769	Ag	1	-
3106.502	Au	-	4.96
3106.96	Ag	1.45	-
3108.822	Au	-	20.97
3108.861	Ag	0.8	-
3109.528	Au	-	11.05
3109.583	Ag	0.31	-
3110.1	Au	-	18.53
3110.129	Ag	0.31	-
3110.208	Ag	0.97	-
3110.439	Au	-	30.92
3112.211	Au	-	3.03

3112.45	Ag	0.37	-
3116.689	Au	-	15.64
3117.09	Ag	0.32	-
3117.558	Ag	0.75	-
3117.623	Au	-	7.72
3118.981	Au	-	22.33
3119.207	Ag	1.3	-
3119.685	Au	-	21.63
3119.734	Ag	0.39	-
3121.657	Au	-	8.4
3122.054	Ag	0.85	-
3123.73	Au	-	10.2
3123.867	Ag	0.98	-
3127.657	Au	-	4.62
3128.186	Au	-	23.24
3128.305	Ag	0.33	-
3128.905	Ag	2.02	-
3129.451	Ag	1.56	-
3129.717	Au	-	6.05
3130.558	Au	-	15.8
3131.545	Ag	2.07	-
3133.159	Au	-	0.87
3134.408	Ag	6.6	-
3134.774	Au	-	10.46
3138.813	Ag	5.54	-

8. References

1. Herman, J.; Harmata, P.; Rychłowicz, N.; Kula, P. Molecular Design of Sexiphenyl-Based Liquid Crystals: Towards Temperature-Stable, Nematic Phases with Enhanced Optical Properties. *Molecules* **2024**, *29*, 946 (1-11).

2. Bruker-Nonius, *SADABS*, Bruker-Nonius, Delft (The Netherlands), **2002**.

3. Altomare, A.; Burla, M. C.; Camalli, M.; Cascarano, G. L.; Giacovazzo, C.; Guagliardi, A.; Moliterni, A. G. G.; Polidori, G.; Spagna, R. *SIR97*: a new tool for crystal structure determination and refinement. *J. Appl. Crystallogr.* **1999**, *32*, 115.

4. Lausi, A. ; Polentarutti, M. ; Onesti, S. ; Plaisier, J. R. ; Busetto, E. ; Bais, G. ; Barba, L. ; Cassetta, A. ; Campi, G. ; Lamba, D. ; Pifferi, A. ; Mande, S. C. ; Sarma, D. D. ; Sharma, S. M. ; Paolucci, G. Status of the crystallography beamlines at Elettra *Eur. Phys. J. Plus* **2015**, *130*, 43.

5. Kabsch, W. *XDS Acta Crystallogr.* **2010**, *D66*, 125.

6. Hübschle, C. B.; Sheldrick, G. M.; Dittrich, B. *ShelXle*: a Qt graphical user interface for *SHELXL. J. Appl. Cryst.* **2011**, *44*, 1281.

7. Sheldrick, G. M. Crystal structure refinement with *SHELXL*. *Acta Crystallogr.* **2015**, *C71*, 3.

8. Farrugia, L. J. *WinGX* and *ORTEP* for *Windows*: an update. *J. Appl. Crystallogr.* **2012**, *45*, 849.

9. Macrae, C. F.; Bruno, I. J.; Chisholm, J. A.; Edgington, P. R.; McCabe, P.; Pidcock, E.; Rodriguez-Monge, L.; Taylor, R.; van de Streek, J.; Wood, P. A. *Mercury CSD 2.0* – new features for the visualization and investigation of crystal structures. *J. Appl. Cryst.* **2008**, *41*, 466.

10. CSD version 5.43 (November 2022) was used in our analysis.

11. Cliffe, M. J.; Goodwin, A. L. *PASCal*: a principal axis strain calculator for thermal expansion and compressibility determination. *J. Appl. Crystallogr.* **2012**, *45*, 1321 (https://www.pascalapp.co.uk/).

12. Erba, A.; Desmarais, J. K.; Casassa, S.; Civalleri, B.; Donà, L.; Bush, I. J.; Searle, B.; Maschio, L.; Edith-Daga, L.; Cossard, A.; Ribaldone, C.; Ascrizzi, E.; Marana, N. L.; Flament, J.-P.; Kirtman, B. *CRYSTAL23*: A Program for Computational Solid State Physics and Chemistry. *J. Chem. Theory Comput.* **2023,** *19*(20), 6891.

13. Perdew, J. P.; Burke, K.; Ernzerhof, M. Generalized Gradient Approximation Made Simple. *Phys. Rev. Lett.* **1996,** *77*(18), 3865.

14. Frisch, M. J.; Pople, J. A.; Binkley, J. S. Self-consistent molecular orbital methods 25., Supplementary functions for Gaussian basis sets. *J. Chem. Phys.* **1984**, *80*(7), 3265.

15. Banks, P. A.; Song, Z.; Ruggiero, M. T. Assessing the Performance of Density Functional Theory Methods on the Prediction of Low-Frequency Vibrational Spectra. *Int. J. Infrared Milli.* **2020**, *41*, 1411–1429.

16. Grimme, S.; Ehrlich, S.; Goerigk, L. Effect of the damping function in dispersion corrected density functional theory. *J. Comput. Chem.* **2011,** *32*(7), 1456-1465.

17. Grimme, S.; Antony, J.; Ehrlich, S.; Krieg, H. A consistent and accurate ab initio parametrization of density functional dispersion correction (DFT-D) for the 94 elements H-Pu. *J. Chem. Phys.* **2010,** *132*(15), 154104.

18. Grimme, S.; Hansen, A.; Brandenburg, J. G.; Bannwarth, C. Dispersion-Corrected Mean-Field Electronic Structure Methods. *Chem. Rev.* **2016,** *116*(9), 5105.

19. Axilrod, B. M.; Teller, E. Interaction of the van der Waals Type Between Three Atoms. *J. Chem. Phys.* **2004,** *11*(6), 299.

20. Ferrero, M.; Rérat, M.; Kirtman, B.; Dovesi, R. Calculation of first and second static hyperpolarizabilities of one- to three-dimensional periodic compounds. Implementation in the *CRYSTAL* code. *J. Chem. Phys.* **2009,** *129*, 244110.

21. Ferrero, M.; Rérat, M.; Orlando, R.; Dovesi, R. Coupled perturbed Hartree-Fock for periodic systems: The role of symmetry and related computational aspects. *J. Chem. Phys.* **2008,** *128*(1), 014110.

22. Ferrero, M.; Rérat, M.; Orlando, R.; Dovesi, R. The calculation of static polarizabilities of 1-3D periodic compounds. the implementation in the *CRYSTAL* code. *J. Comput. Chem.* **2008,** *29*(9), 1450.

23. Juneja, N.; Hastings, J. L.; Stoll, W. B.; Brennessel, W. W.; Zarrella, S.; Sornberger, P.; Catalano, L.; Korter, T. M.; Ruggiero, M. T. Fundamentally intertwined: anharmonic intermolecular interactions dictate both thermal expansion and terahertz lattice dynamics in molecular crystals. *Chem. Commun.* **2024**, *60*, 12169-12172.

24. *Jmol*: an open-source *Java* viewer for chemical structures in 3D. http://www.jmol.org/ .
